# Supplementary figures and images for: STIL overexpression shortens lifespan and reduces tumor formation in mice
Source: PLoS Genet. 2024 Oct 28;20(10):e1011460. doi: 10.1371/journal.pgen.1011460 (PMC11542878; doi:10.1371/journal.pgen.1011460)

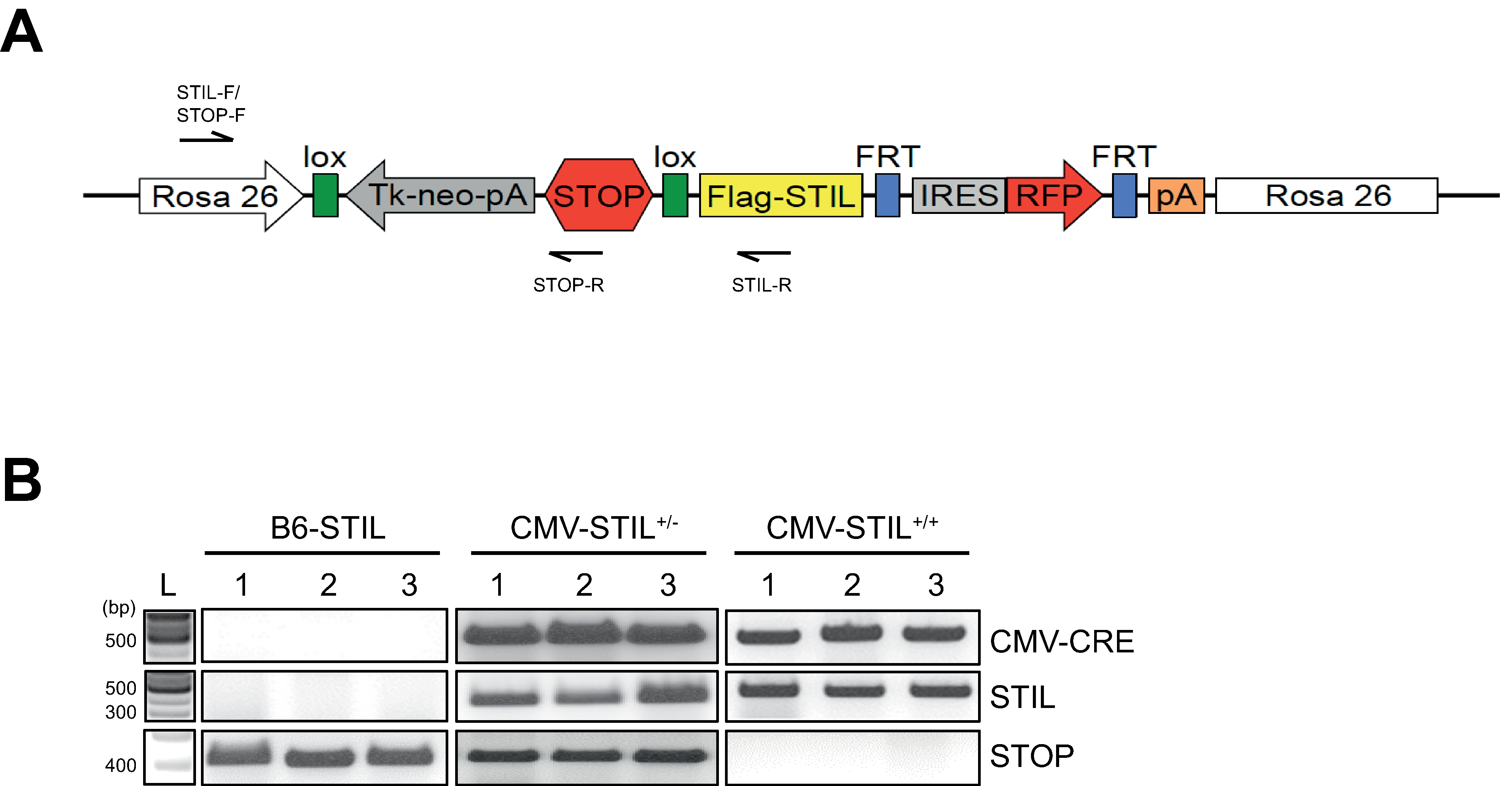

Supplement: S1 Fig — (A) Schematic representation of the loxP-STOP-loxP-FLAG-STIL transgene. The localization of primers used for genotyping is indicated by arrows. (B) Genotyping of B6-STIL, CMV-STIL+/- and CMV-STIL+/+ MEFs. For each MEF line three independent clones are shown. B6-STIL control MEFs lack CMV-CRE, whereas CMV-STIL+/- and CMV-STIL+/+ MEFs are CMV-CRE positive. The FLAG-STIL transgene with excised loxP-STOP-loxP cassette is present in CMV-STIL+/- and CMV-STIL+/+ but not B6-STIL MEFs. Biallelic loss of the loxP-STOP-loxP cassette in CMV-STIL+/+ MEFs is verified by the absence of a STOP cassette PCR product. L, DNA ladder. (TIF) [file pgen.1011460.s001.tif]

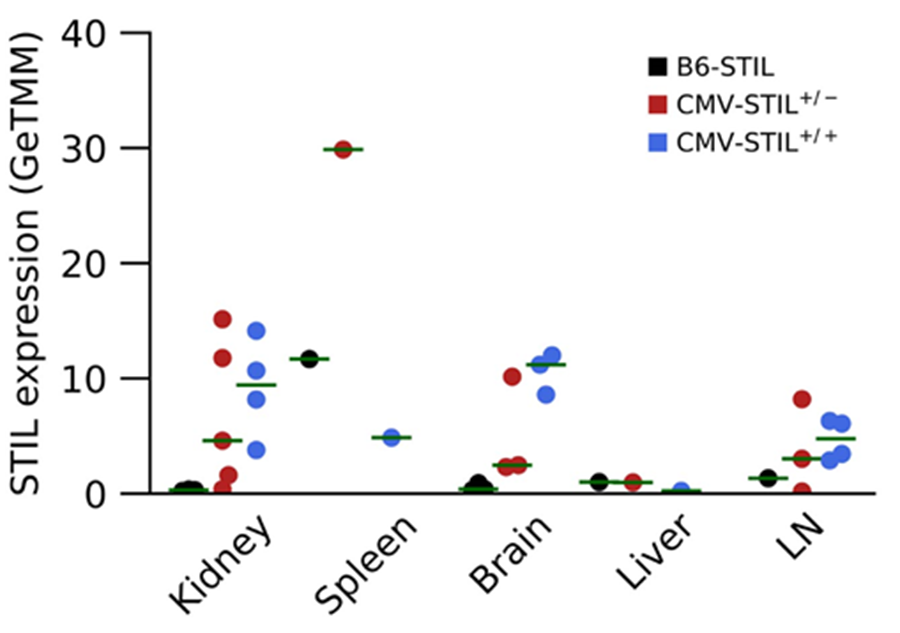

Supplement: S2 Fig — RNA sequencing showing STIL mRNA levels in different normal organs from B6-STIL control, CMV-STL+/- and CMV-STL+/+ mice. (TIF) [file pgen.1011460.s002.tif]

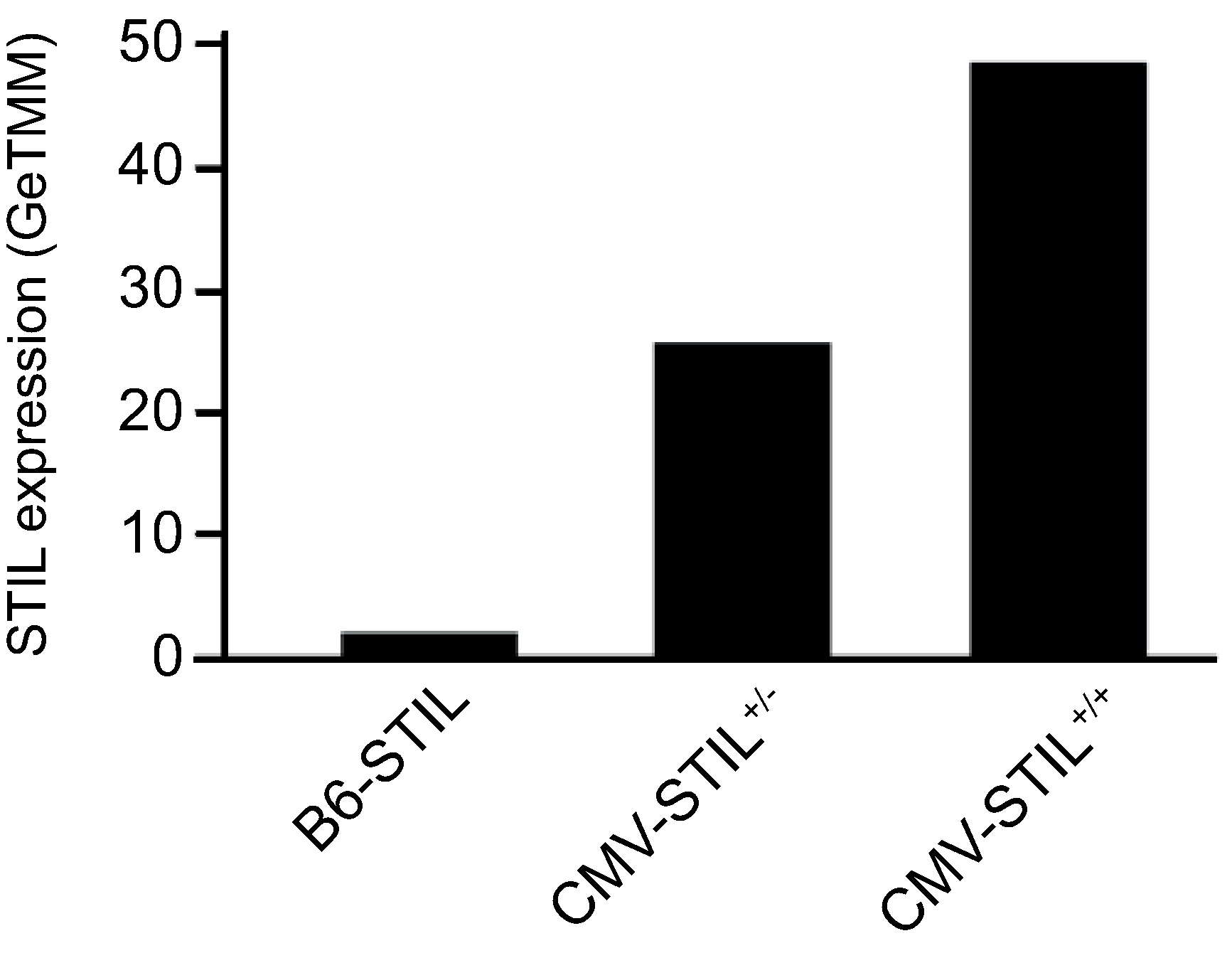

Supplement: S3 Fig — RNA sequencing showing STIL mRNA levels in MEFs (p3) from B6-STIL control, CMV-STL+/- and CMV-STL+/+ mice. (TIF) [file pgen.1011460.s003.tif]

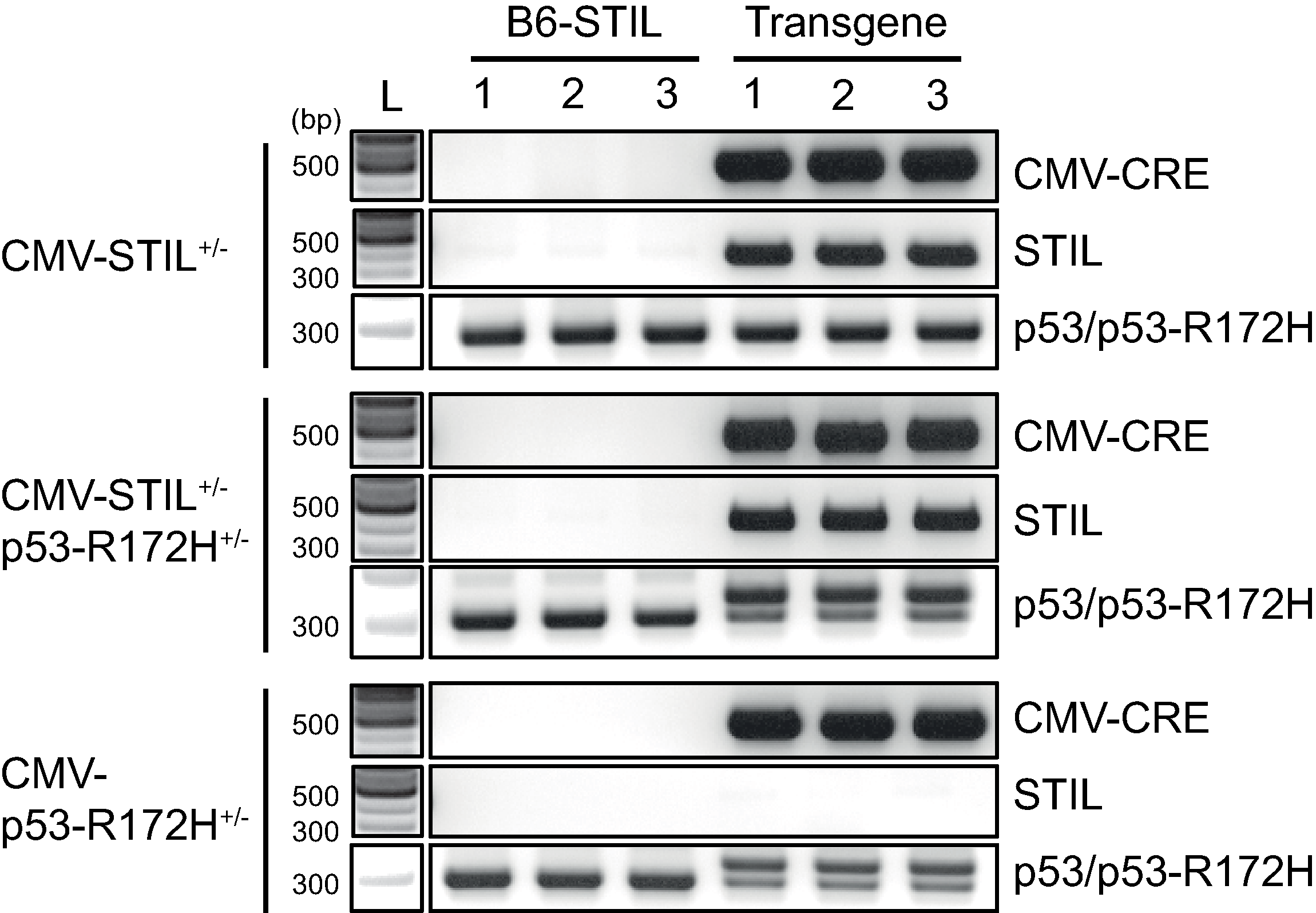

Supplement: S4 Fig — For each MEF line three independent clones are shown. B6-STIL control MEFs are negative for CMV-CRE and the STIL transgene with excised loxP-STOP-loxP cassette, and harbor only wildtype TP53. CMV-STIL+/- and CMV-p53-R172H+/- MEFs are both positive for CMV-CRE and the STIL transgene, and harbor only wildtype TP53 as well. CMV-p53-R172H+/- MEFs in addition harbor a mutant TP53-R172H allele (double band). CMV-p53-R172H+/- MEFs are positive for CMV-CRE but negative for the STIL transgene, and harbor both wildtype and mutant TP53. L, DNA ladder. (TIF) [file pgen.1011460.s004.tif]

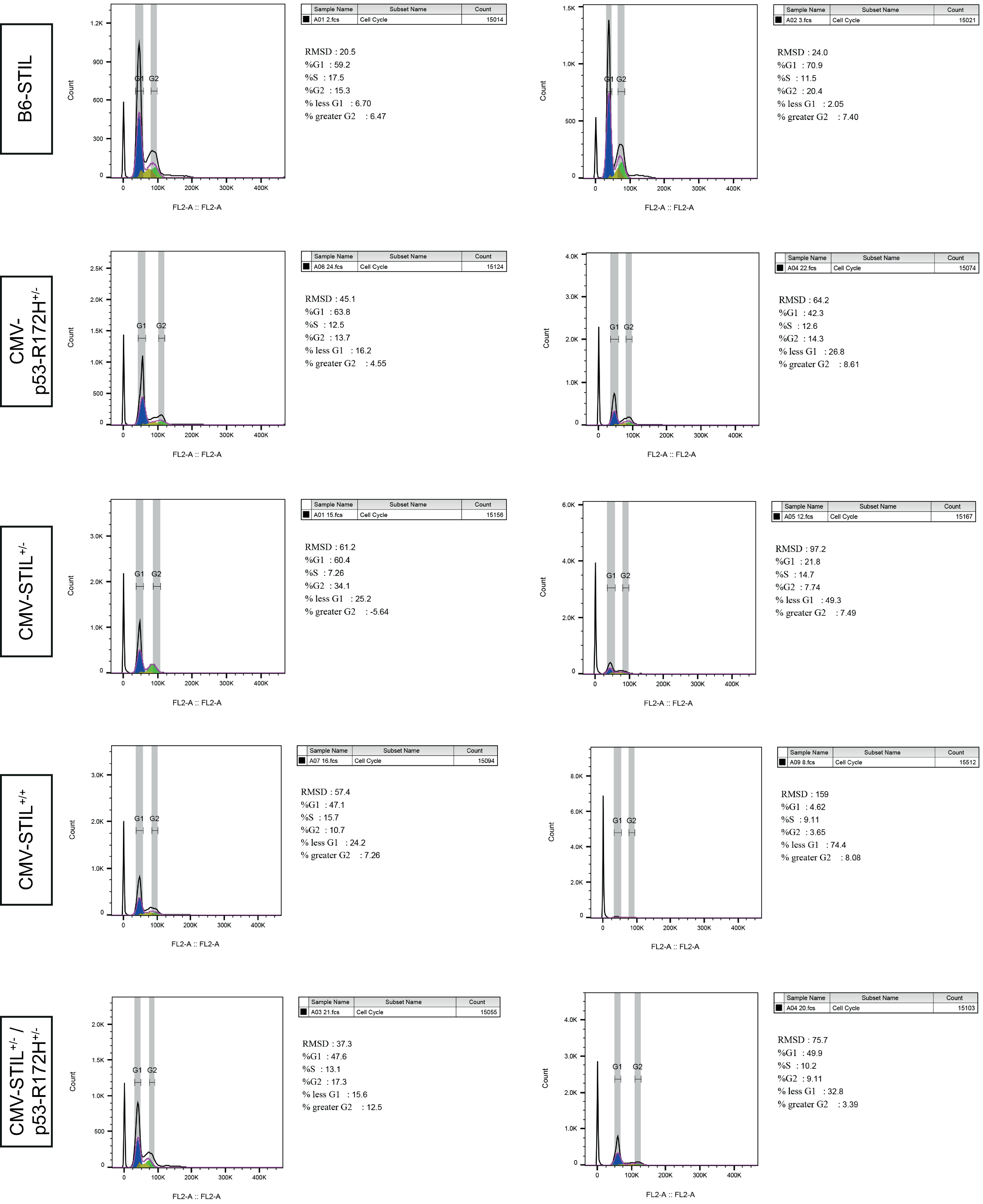

Supplement: S5 Fig — Two representative examples of fluorescence activated cell sorting (FACS) profiles of cell cycle analyses after propidium iodide staining per genotype are shown. Percentages of cells per cell cycle phase and sub-G1 phase, apoptotic cells are given for B6-STIL control, CMV-p53-R172H+/-, CMV-STIL+/-, CMV-STIL+/+, and CMV-STIL+/-/p53-R172H+/- MEFs. (TIF) [file pgen.1011460.s005.tif]

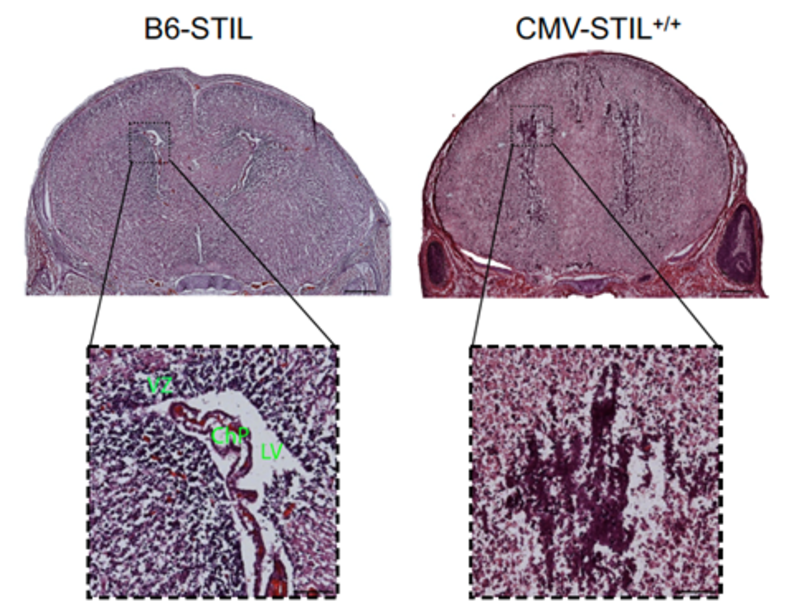

Supplement: S6 Fig — In contrast to brains from B6-STIL control mice at postnatal day 0 (left upper panel), the lateral ventricles (boxed regions in upper panels) appear to be collapsed in CMV-STIL+/+ animals (right upper panel). The boxed regions in the upper panels are shown enlarged in insets (lower panels). VZ, ventricular zone; LV, lateral ventricle; ChP, choroid plexus. Scale bars in upper panels, 500 μm; Scale bars in insets, 100 μm. (TIF) [file pgen.1011460.s006.tif]

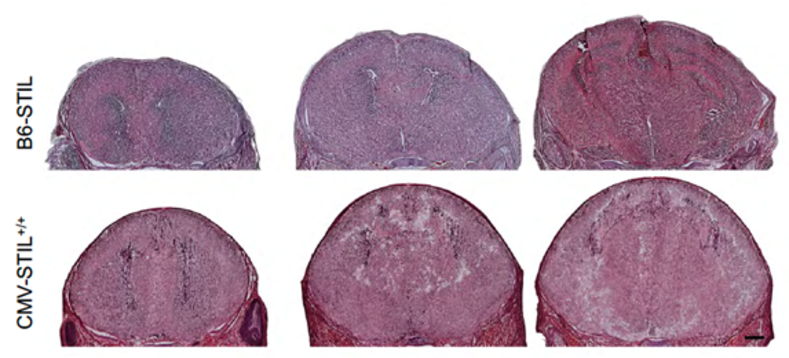

Supplement: S7 Fig — Serial sectioning through the anterior/posterior extent of the brain fails to reveal a clearly defined lateral ventricle in postnatal day 0 CMV-STIL+/+ animals (lower panel). For comparison serial sections from a postnatal day 0 B6-STIL control mouse brain (upper panel) clearly depicting lateral ventricles are shown in the upper panel. (TIF) [file pgen.1011460.s007.tif]

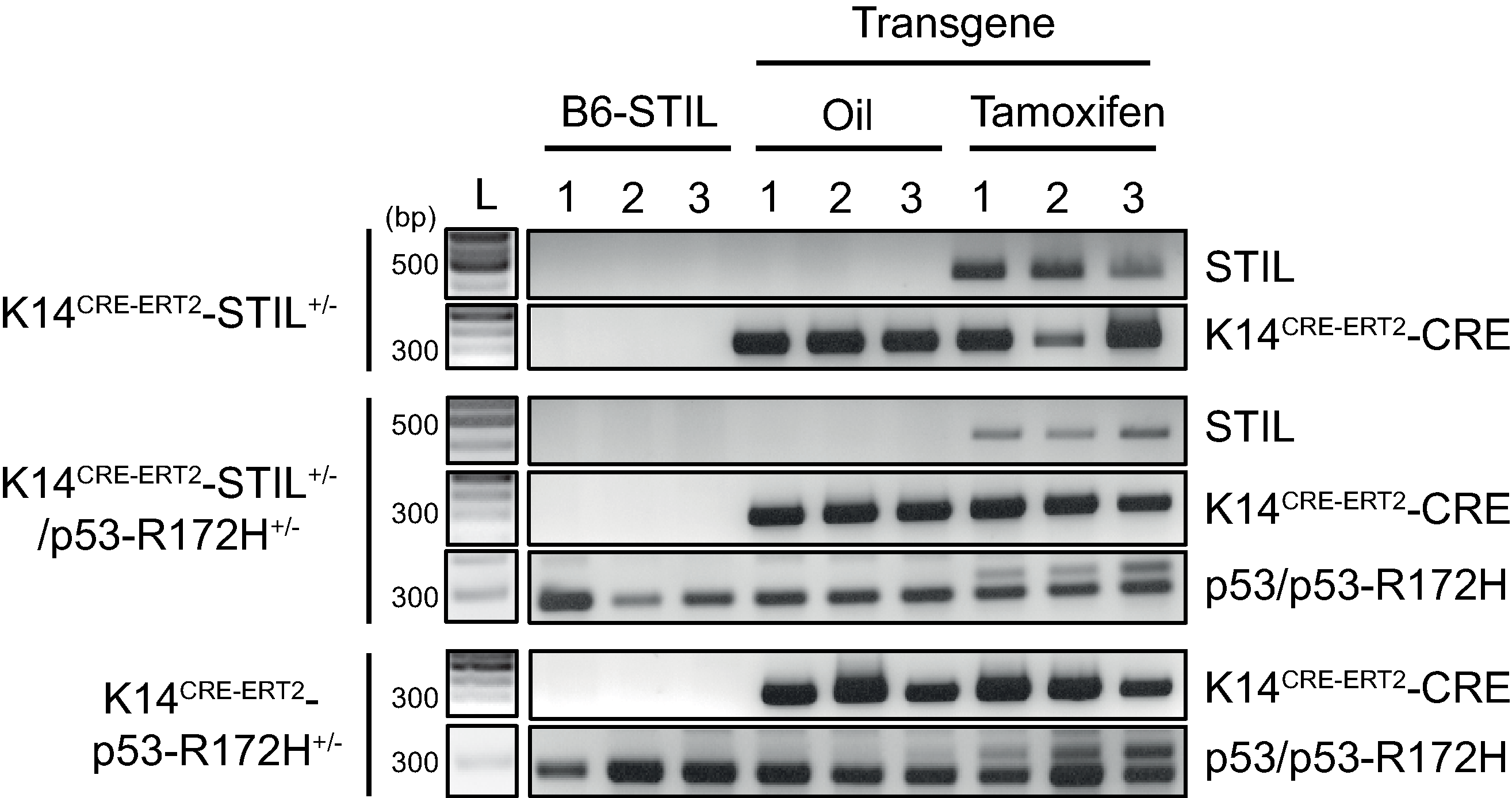

Supplement: S8 Fig — Genotyping of B6-STIL, and oil- versus tamoxifen-treated K14CRE-ERT2-STIL+/-, K14CRE-ERT2-STIL+/-/p53-R172H+/- and K14CRE-ERT2-p53-R172H+/- mice (n = 3 for each condition). The three B6-STIL mice are negative for K14CRE-ERT2-CRE and the STIL transgene, and harbor only wildtype TP53. K14CRE-ERT2-STIL+/- and K14CRE-ERT2-STIL+/-/p53-R172H+/- mice are positive for the STIL transgene, and K14CRE-ERT2-STIL+/-/p53-R172H+/- and K14CRE-ERT2-p53-R172H+/- mice for mutant TP53 only after tamoxifen treatment. L, DNA ladder. (TIF) [file pgen.1011460.s008.tif]

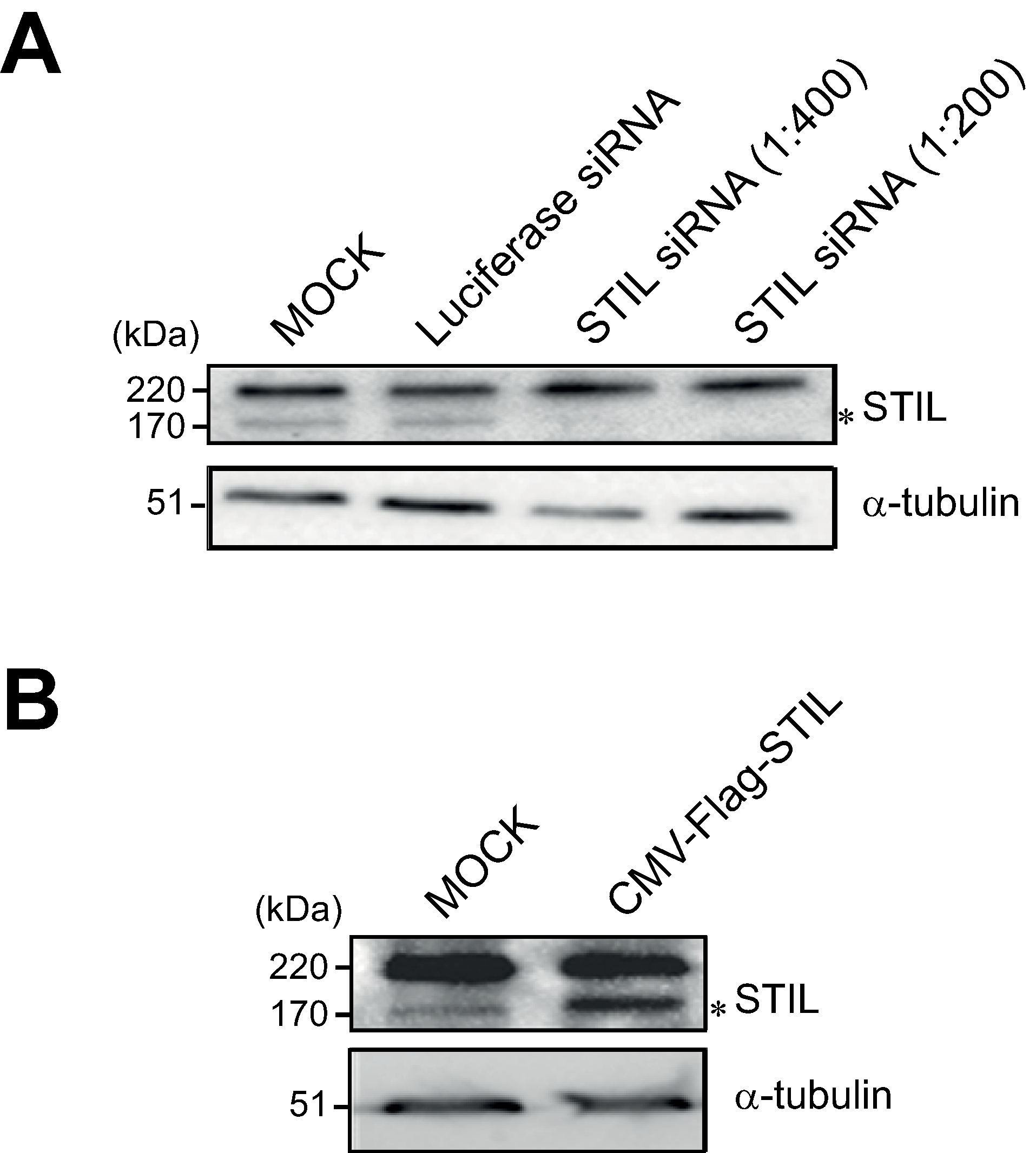

Supplement: S9 Fig — (A) MEFs were transfected with control luciferase or STIL siRNA and immunoblotted with a rabbit anti-STIL antibody (A302-442A, Bethyl Laboratories) that detects human and mouse STIL at a size of 170 kDa (asterisk). siRNA-mediated knockdown of STIL led to the specific disappearance of the 170 kDa band. (B) Immunoblotting of lysates from MEFs transiently transfected with a CMV-Flag-STIL expression plasmid specifically enhanced the 170 kDa band when probed with the rabbit anti-STIL antibody (A302-442A, Bethyl Laboratories). (TIF) [file pgen.1011460.s009.tif]
