# Supplementary material for: STIL overexpression shortens lifespan and reduces tumor formation in mice
Source: PLoS Genet. 2024 Oct 28;20(10):e1011460. doi: 10.1371/journal.pgen.1011460 (PMC11542878; doi:10.1371/journal.pgen.1011460)
Supplement: S1 Table — (DOCX) [file pgen.1011460.s010.docx]

**S1Table.** Primers for genotyping and qPCR.

|  | **Name** | **Forward primer (5' - 3')** | **Reverse primer (5' - 3')** |
| --- | --- | --- | --- |
| **genotyping** | STOP | AAAGTCGCTCTGAGTTGTTAT | GTGGCAGCTTCTTTAGCAAC |
|  | STIL | AAAGTCGCTCTGAGTTGTTAT | CATCGTCGTCCTTGTAGTCAG |
|  | CMV-CRE | GGCGCGGCAACACCATTTTT | CCGGGCTGCCACGACCAA |
|  | K14-CRE | CGCCAATTAACCCTCACTAAAGG | ATCCATCAAATCGACCACCA |
|  | TP53 | AGCCTGCCTAGCTTCCTCAGG | CTTGGAGACATAGCCACACTG |
| **qPCR** | STIL_qPCR | TCCTTGTGAGAGTAGGACGC | TCAAGGTCAGTGTCATGCTT |
|  | HPRT | TGATCAGTCAACGGGGGACA | TTCGAGAGGTCCTTTTCACCA |
|  | PBIB | TCGTCTTTGGACTCTTTGGAA | AGCGCTCACCATAGATGCTC |
