# Supplementary material for: STIL overexpression shortens lifespan and reduces tumor formation in mice
Source: PLoS Genet. 2024 Oct 28;20(10):e1011460. doi: 10.1371/journal.pgen.1011460 (PMC11542878; doi:10.1371/journal.pgen.1011460)
Supplement: S2 Table — (DOCX) [file pgen.1011460.s011.docx]

**S2 Table.** Primary antibodies for immunoblotting (IB) and immuno-fluorescence (IF).

|  | **Antibody** | **Clone** | **Company** | **Cat. No.** | **Dilution** | |
| --- | --- | --- | --- | --- | --- | --- |
| **IB** | rabbit anti-STIL |  | Bethyl Laboratories | A302-442A | 1:1000 |  |
|  | mouse-β-actin-HRP | C4 | Santa Cruz | sc-47778 HRP | 1:5000 |  |
|  | mouse-α-tubulin | DM1A | Sigma | T6199 | 1:5000 |  |
|  | mouse-p16^INK4A^ | F-4 | Santa Cruz | sc-74401 | 1:500 |  |
| **IF** | mouse-anti-centrin | 20H5 | Millipore | 1624 | 1:1000 |  |
|  | rabbit-anti-pericentrin |  | Abcam | ab4448 | 1:1000 |  |
|  | rabbit-anti-Ki67 | D3B5 | Cell Signaling | 9129T | 1:100 |  |
|  | rabbit-anti-CD20 |  | Proteintech |  |  |  |
