# Supplementary material for: STIL overexpression shortens lifespan and reduces tumor formation in mice
Source: PLoS Genet. 2024 Oct 28;20(10):e1011460. doi: 10.1371/journal.pgen.1011460 (PMC11542878; doi:10.1371/journal.pgen.1011460)

Ladder = Precision Plus Protein Standards Dual Color (Bio-Rad)

Figure 1B

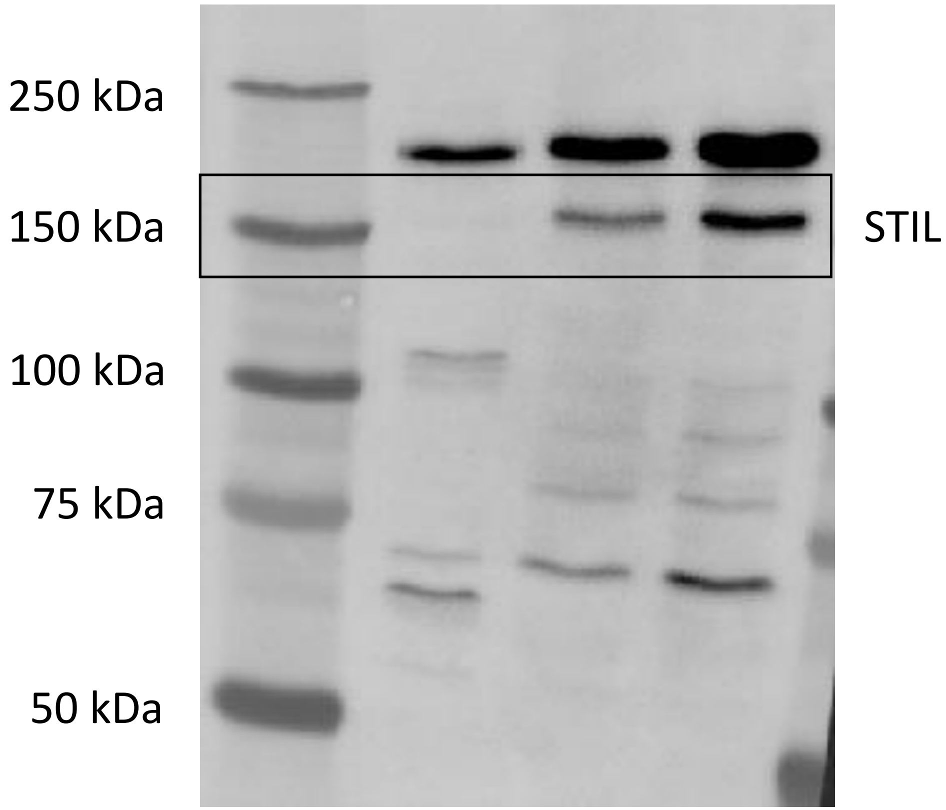

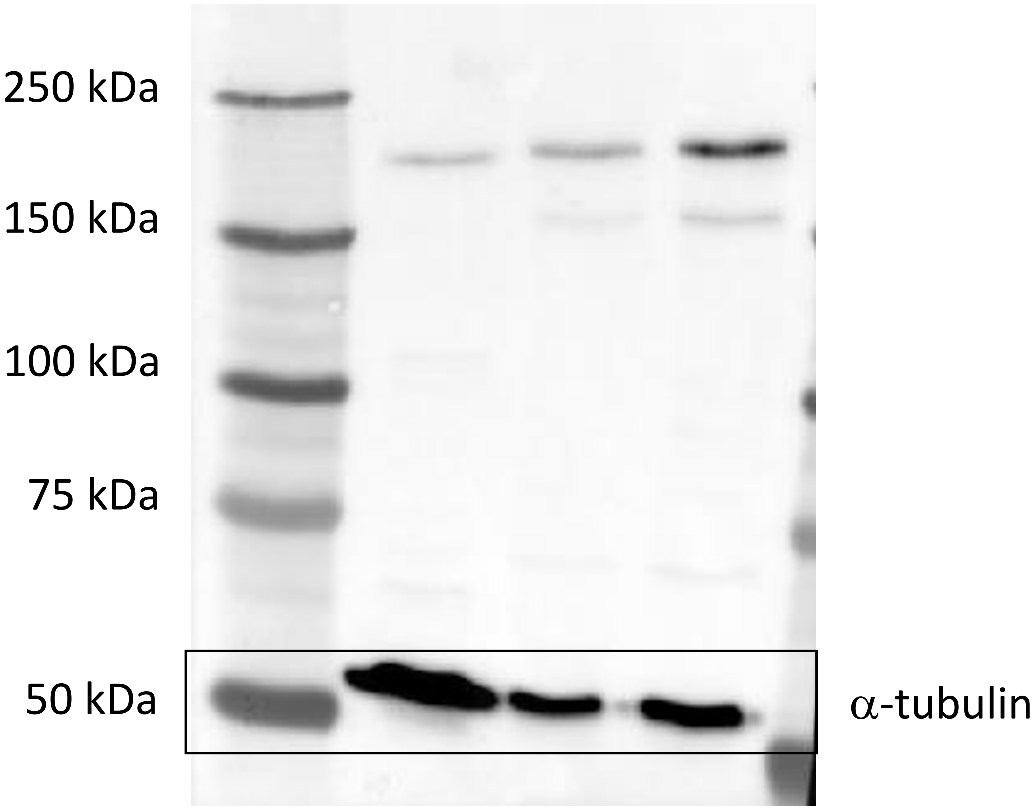

Ladder = Precision Plus Protein Standards Dual Color (Bio-Rad)

Figure 3B

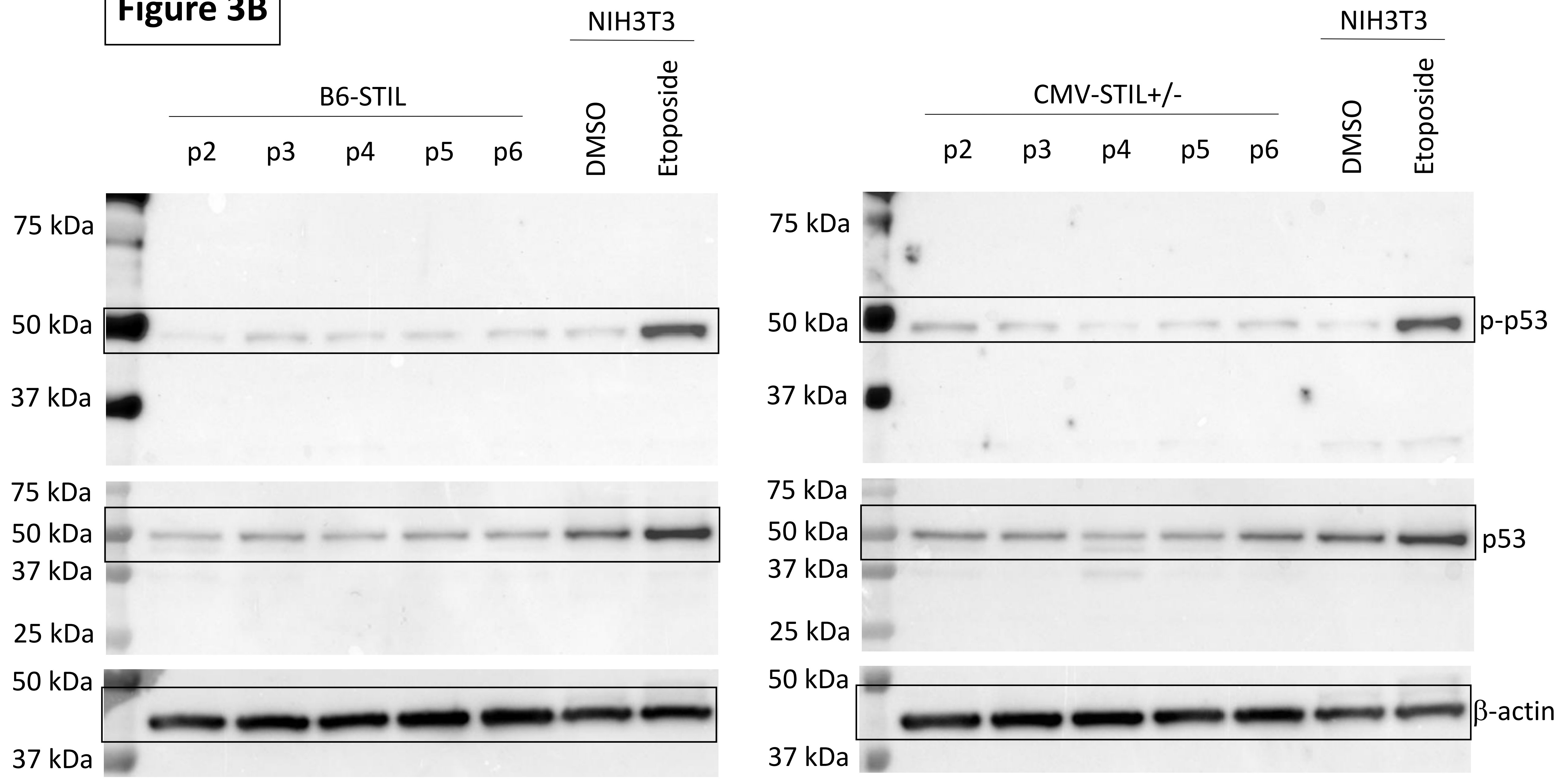

Ladder = Precision Plus Protein Standards Dual Color (Bio-Rad)

Figure 3E

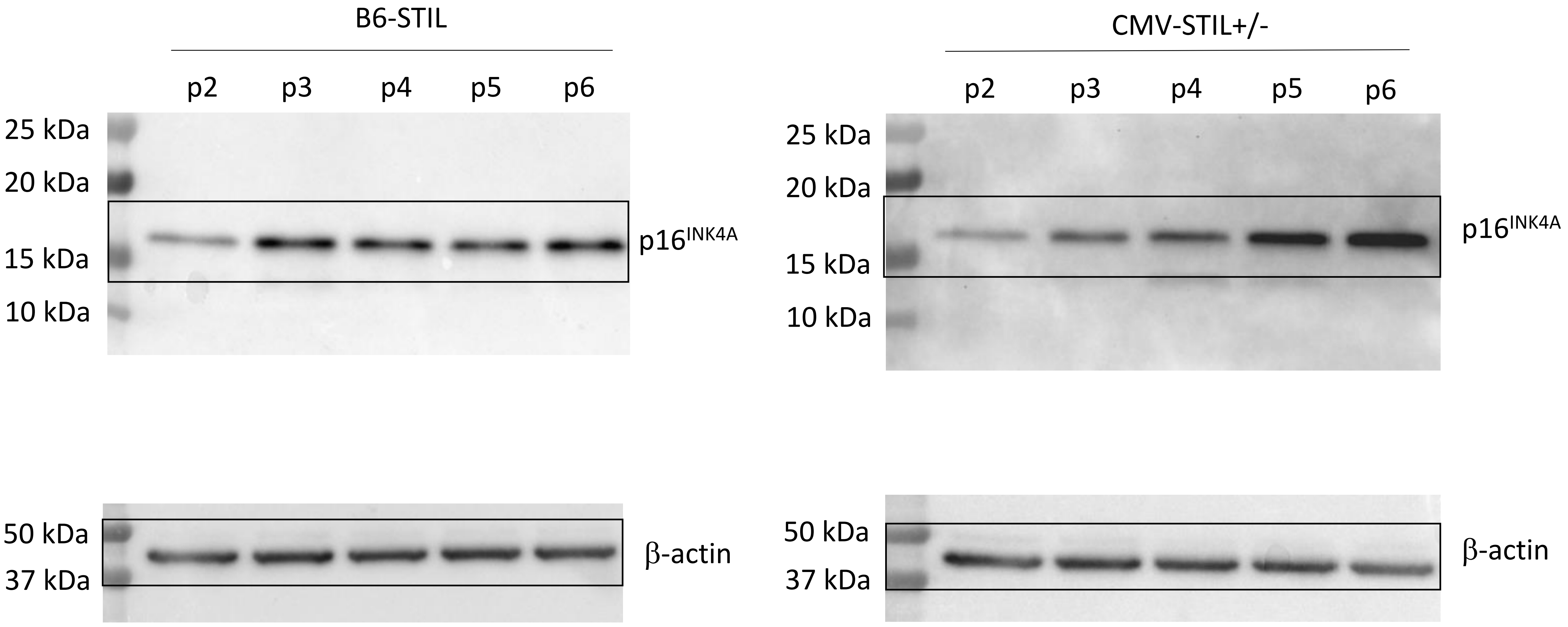

Figure 3K

Ladder = Precision Plus Protein Standards Dual Color (Bio-Rad)

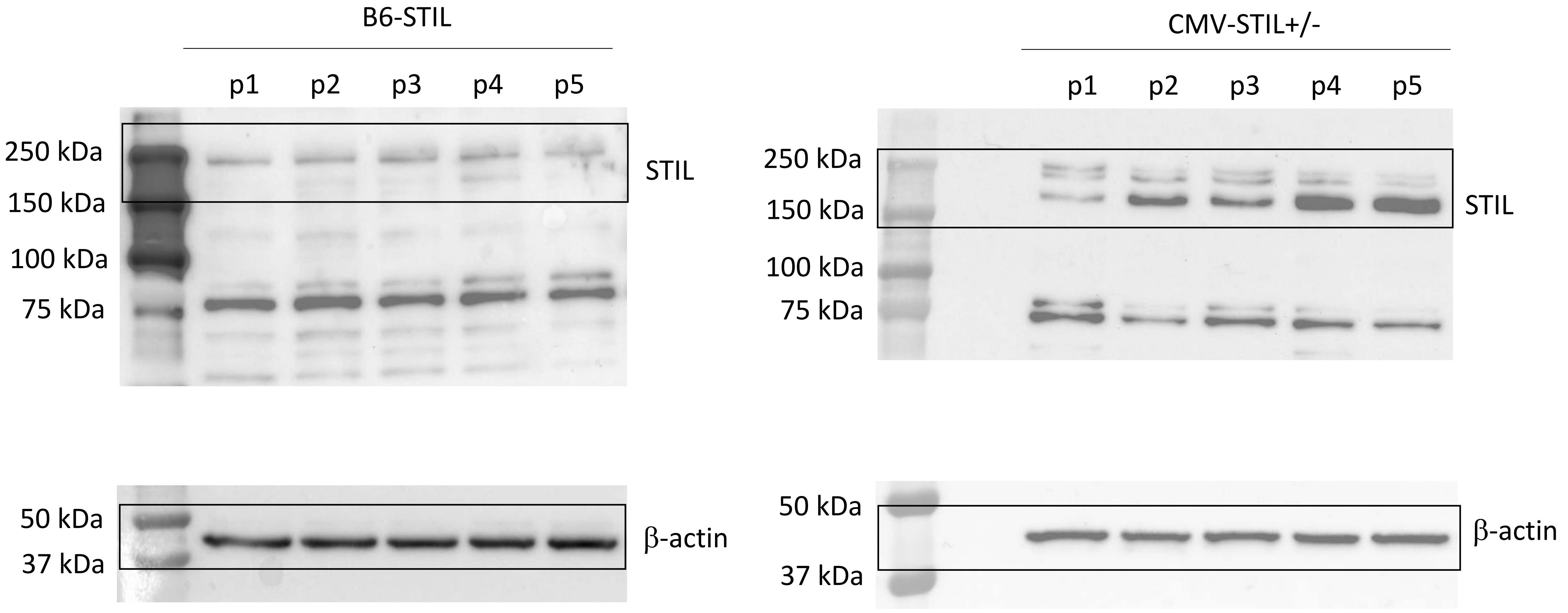

Ladder = Precision Plus Protein Standards Dual Color (Bio-Rad)

Figure 4G

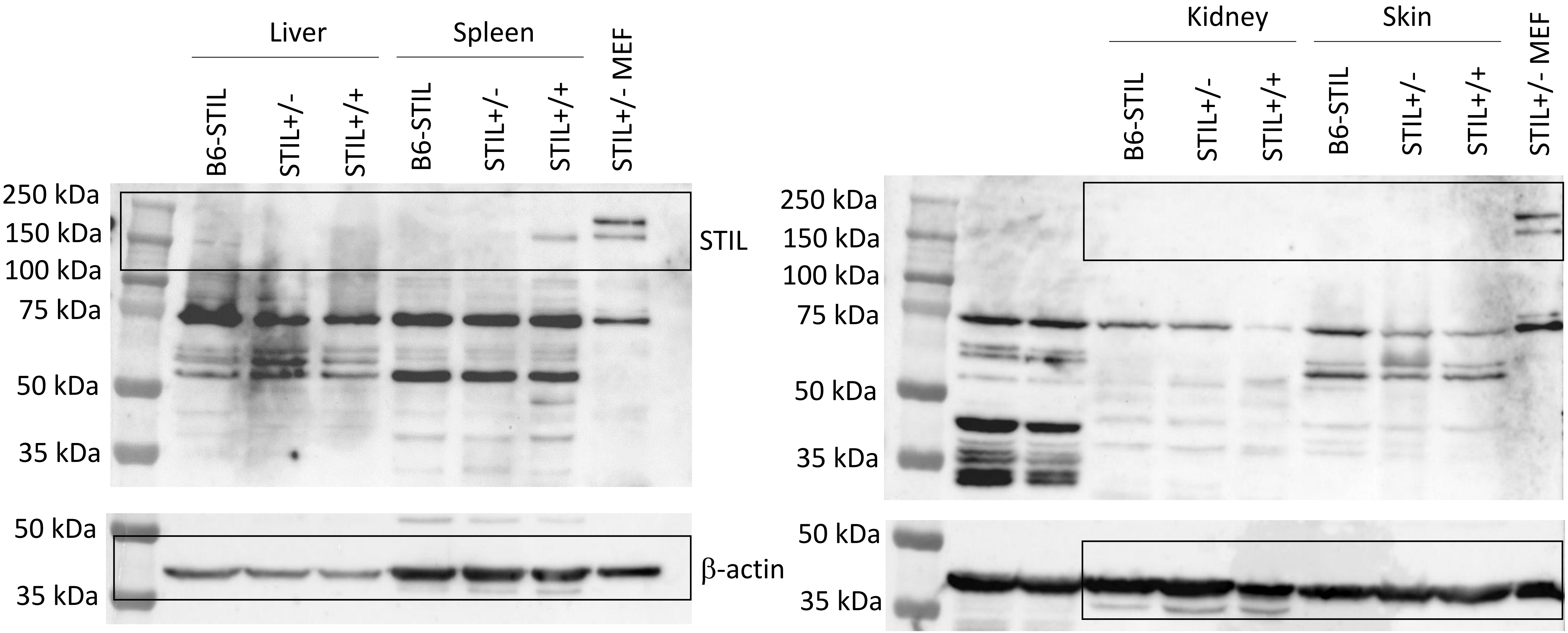

Ladder = Precision Plus Protein Standards Dual Color (Bio-Rad)

Figure 5E

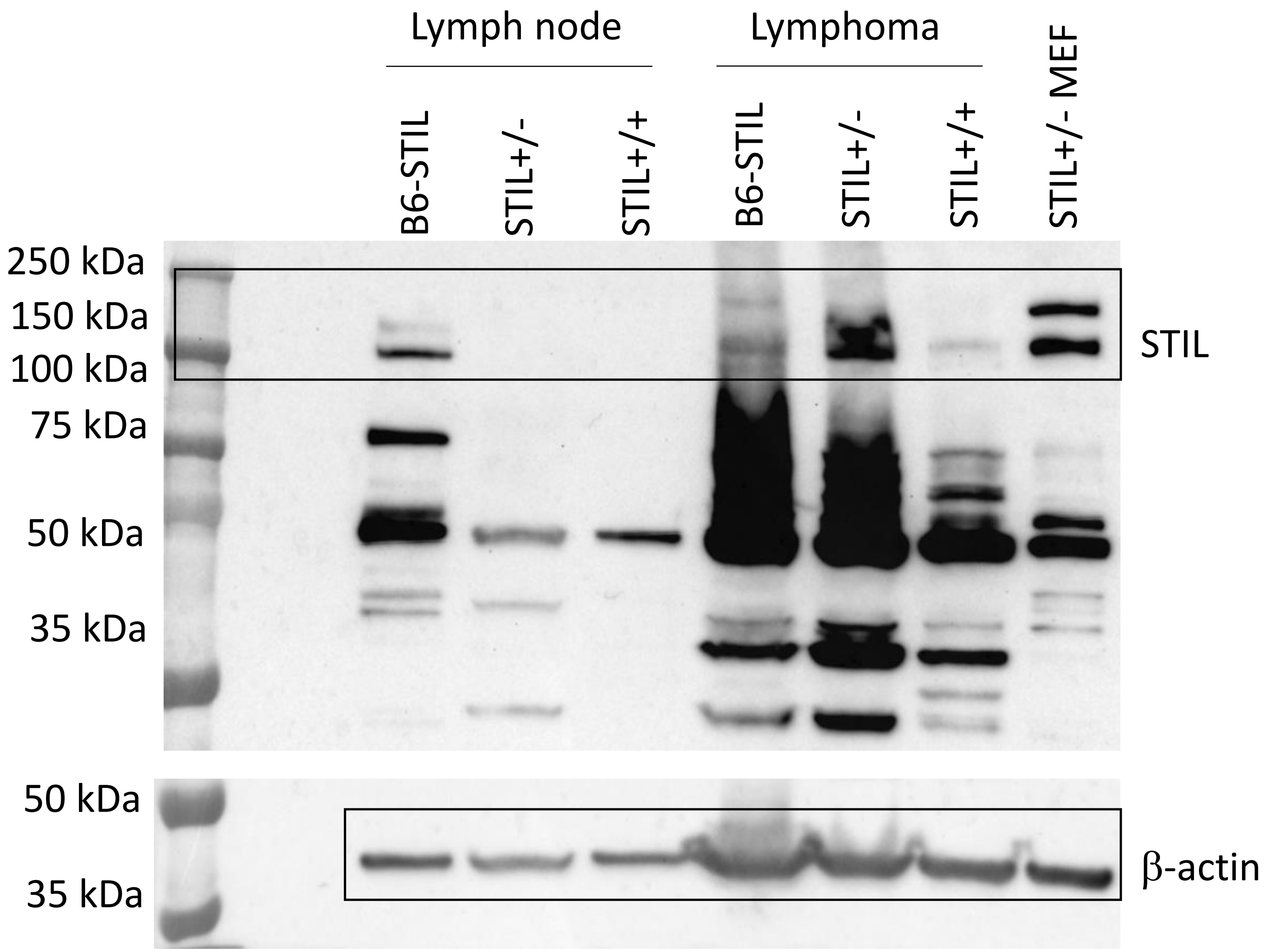

Ladder = Precision Plus Protein Standards Dual Color (Bio-Rad)

Supplemental Figure S9

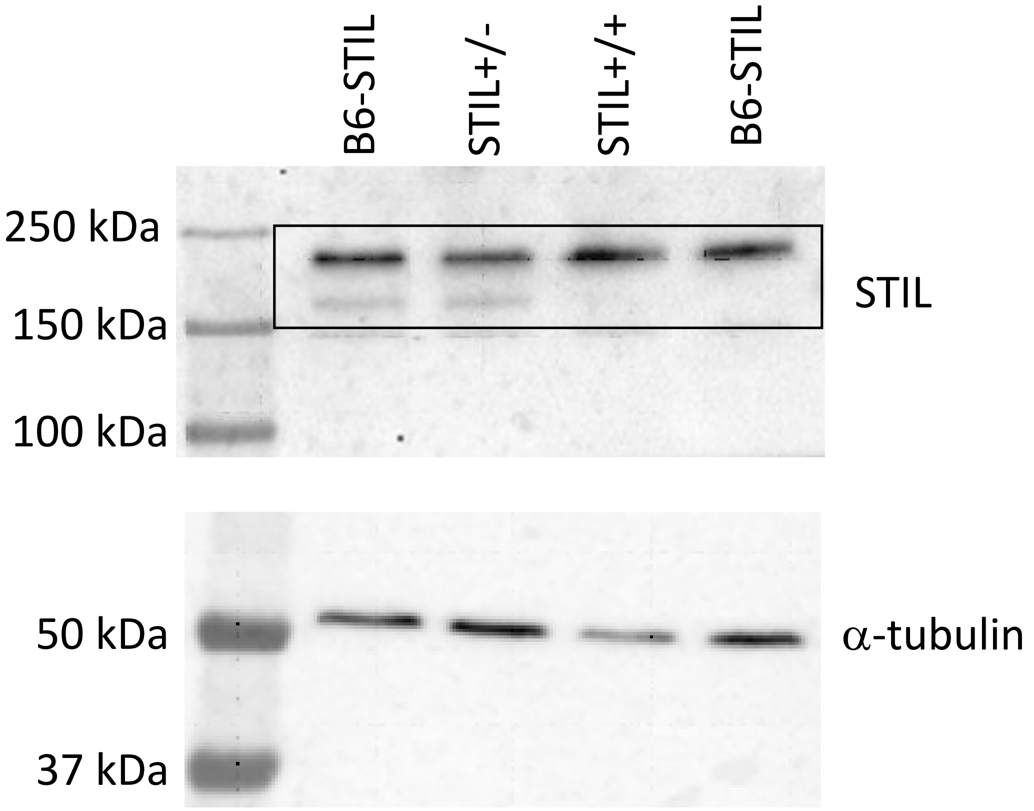

Supplement: S1 Appendix — (PDF) [file pgen.1011460.s012.pdf]
