# Supplementary material for: STIL overexpression shortens lifespan and reduces tumor formation in mice
Source: PLoS Genet. 2024 Oct 28;20(10):e1011460. doi: 10.1371/journal.pgen.1011460 (PMC11542878; doi:10.1371/journal.pgen.1011460)
Supplement: S2 Appendix — (PDF) [file pgen.1011460.s013.pdf]

Figure 3H

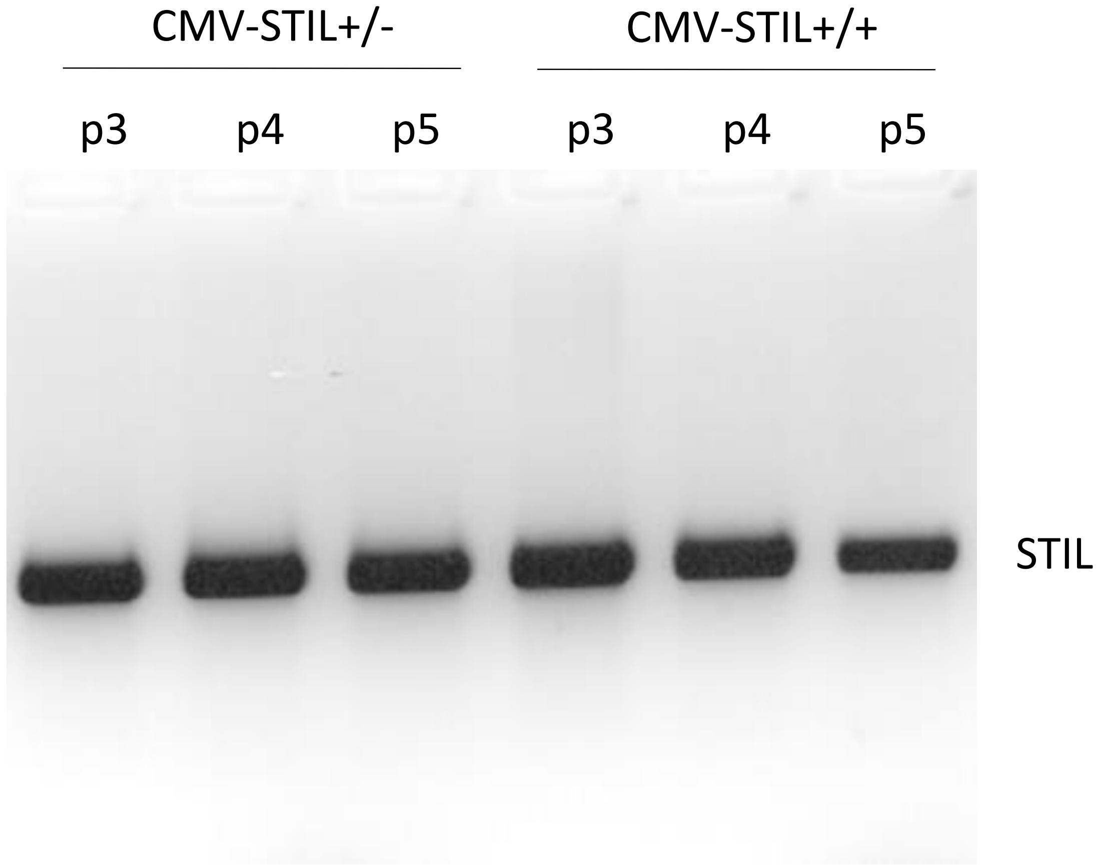

Supplemental Figure S1B

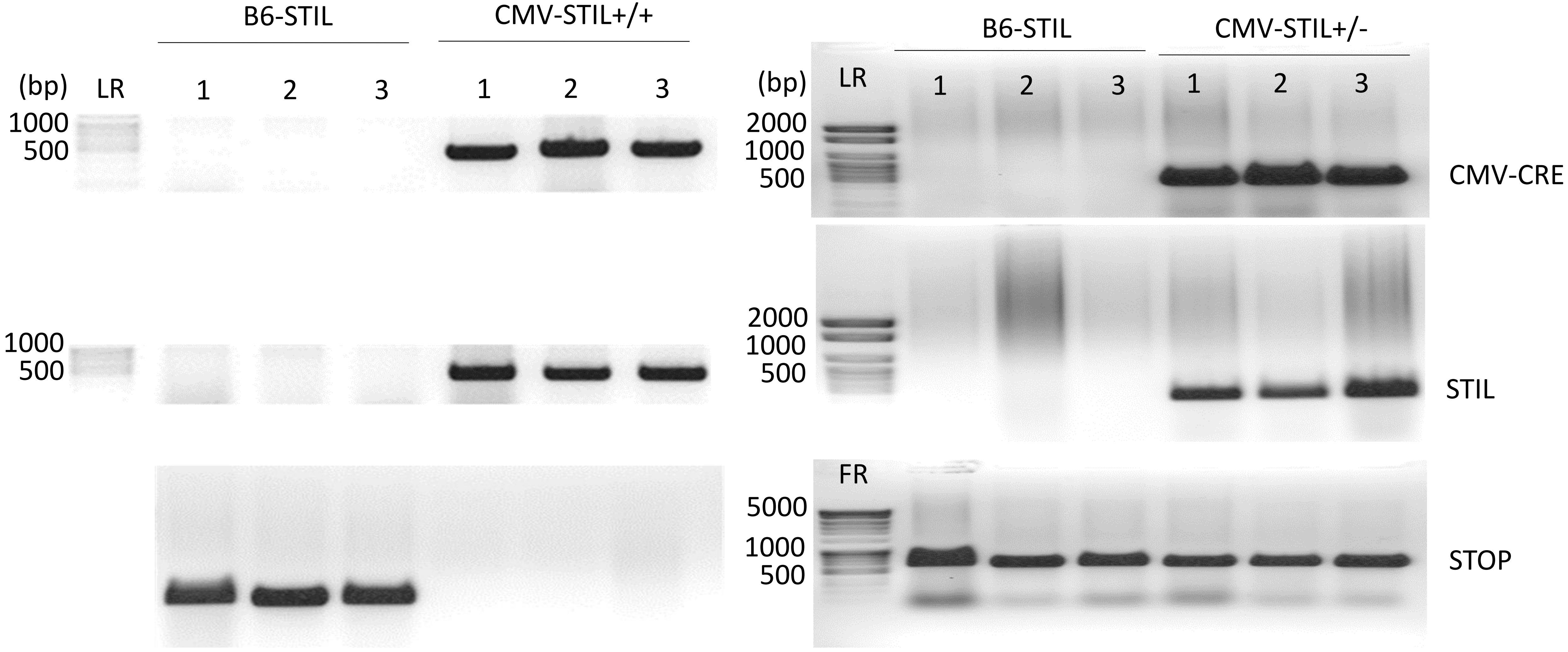

LR = LowRanger 100 bp DNA Ladder (Norgen Biotek Corp.)

FR = FullRanger 100 bp DNA Ladder (Norgen Biotek Corp.)

Supplemental Figure S4

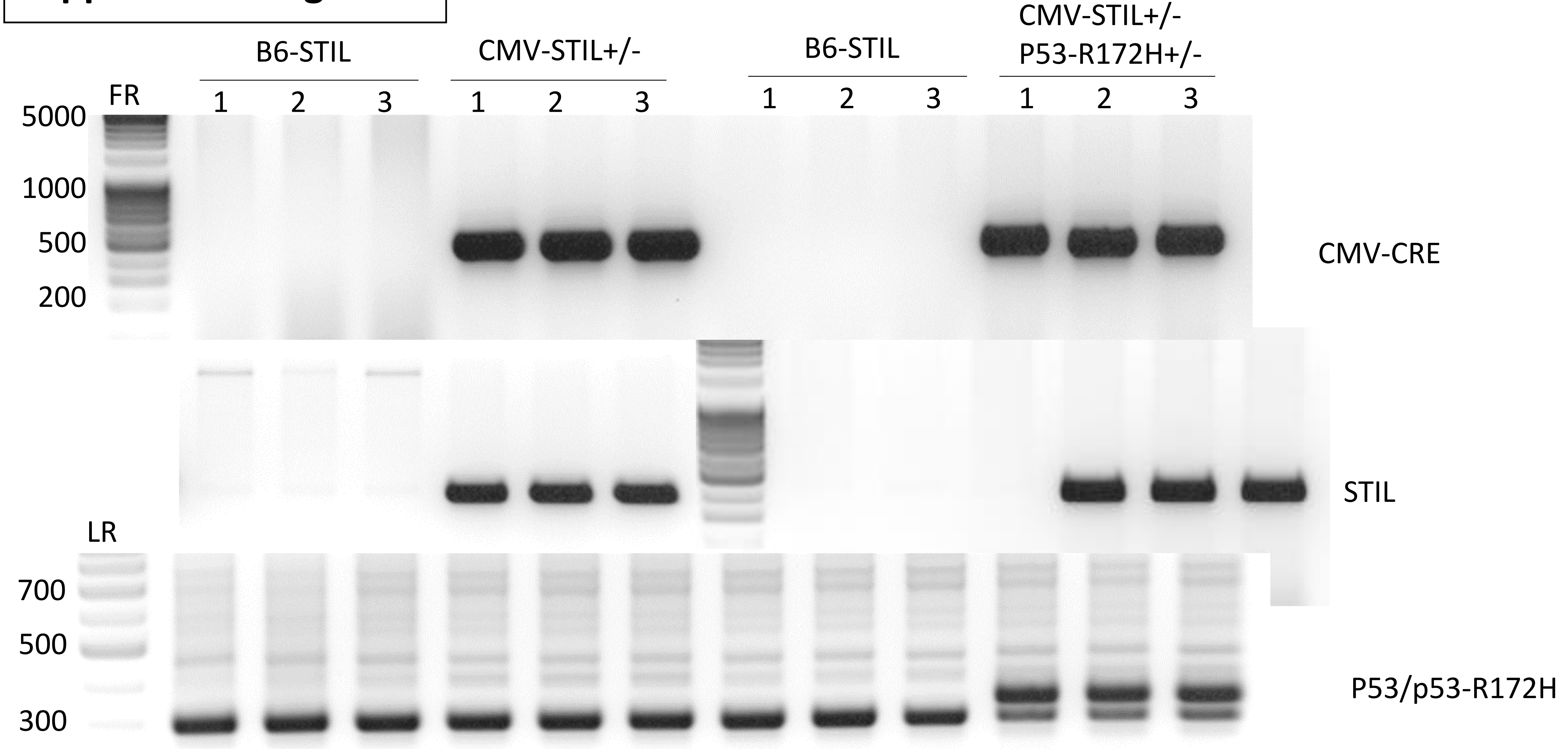

LR = LowRanger 100 bp DNA Ladder (Norgen Biotek Corp.) FR = FullRanger 100 bp DNA Ladder (Norgen Biotek Corp.)

Supplemental Figure S4

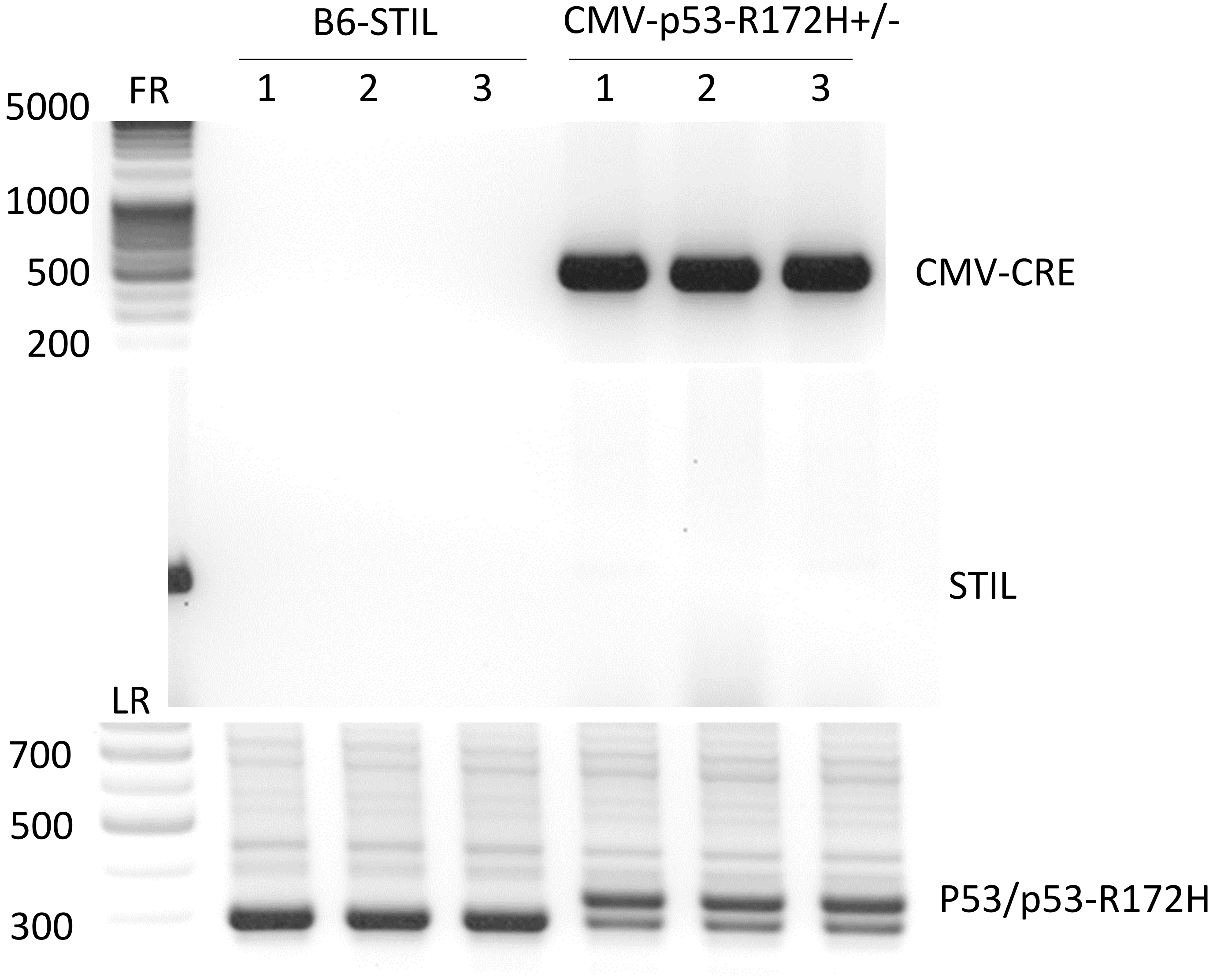

LR = LowRanger 100 bp DNA Ladder (Norgen Biotek Corp.)

FR = FullRanger 100 bp DNA Ladder (Norgen Biotek Corp.)

Supplemental Figure S8

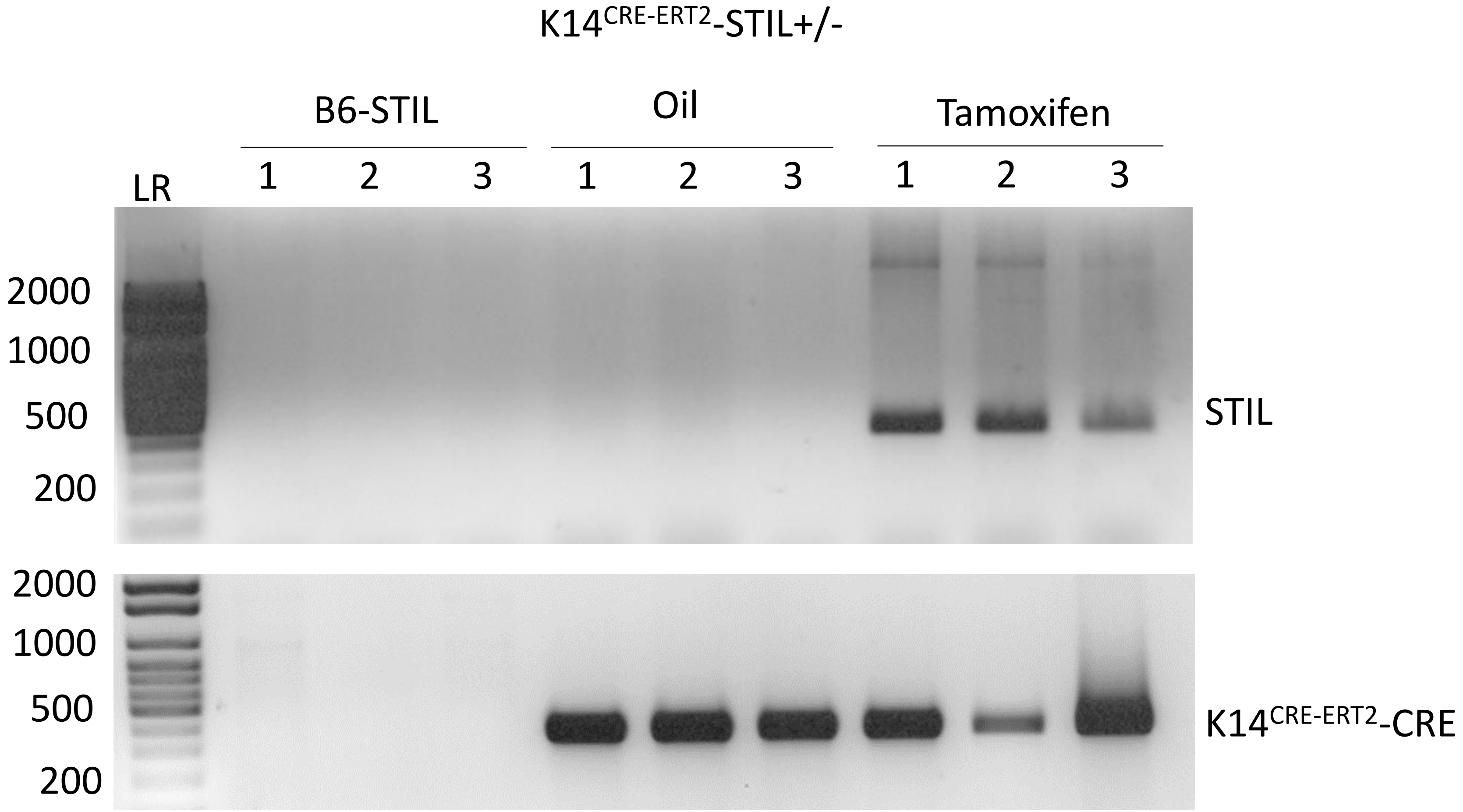

LR = LowRanger 100 bp DNA Ladder (Norgen Biotek Corp.)

Supplemental Figure S8

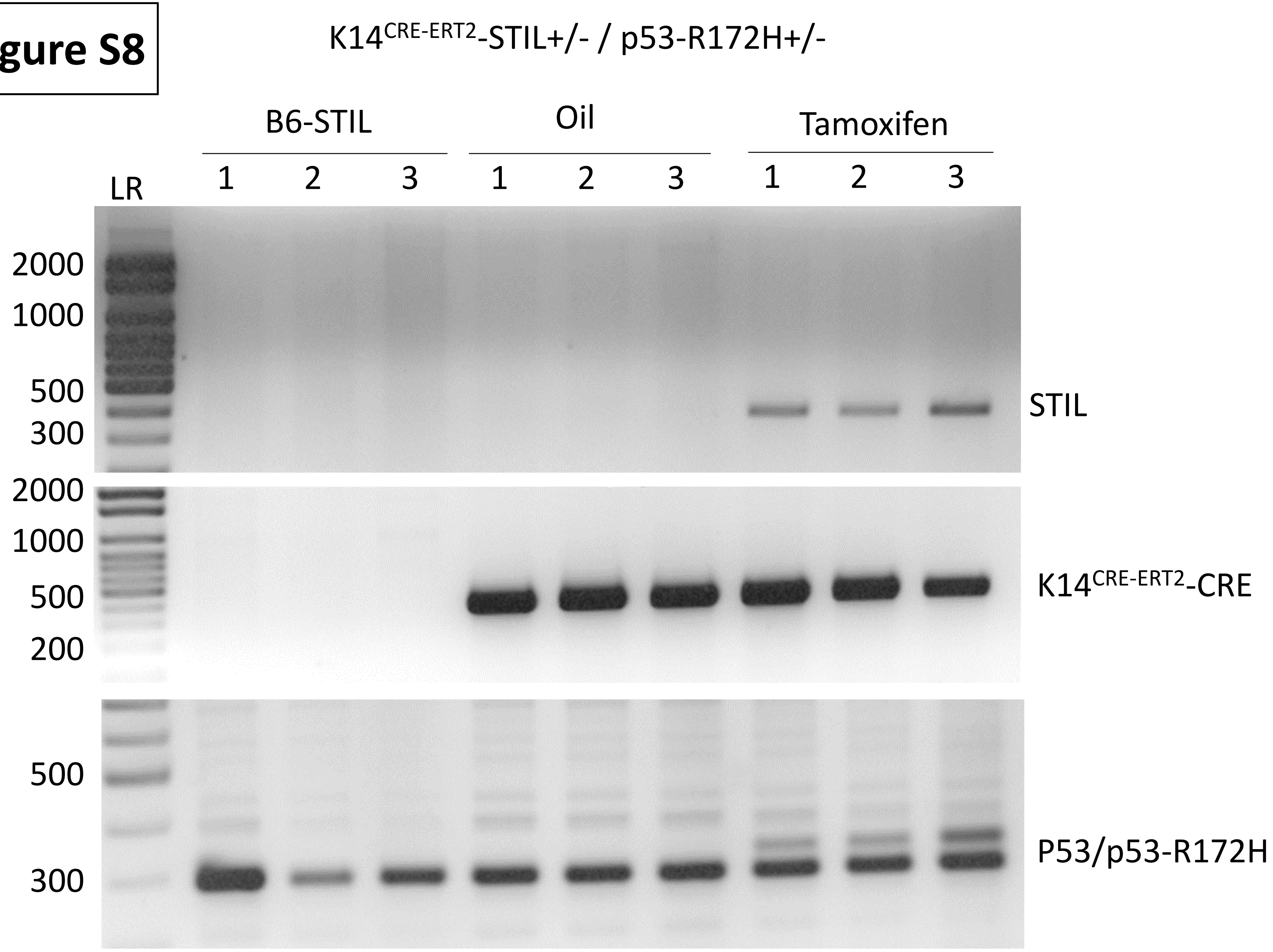

LR = LowRanger 100 bp DNA Ladder (Norgen Biotek Corp.)

Supplemental Figure S8

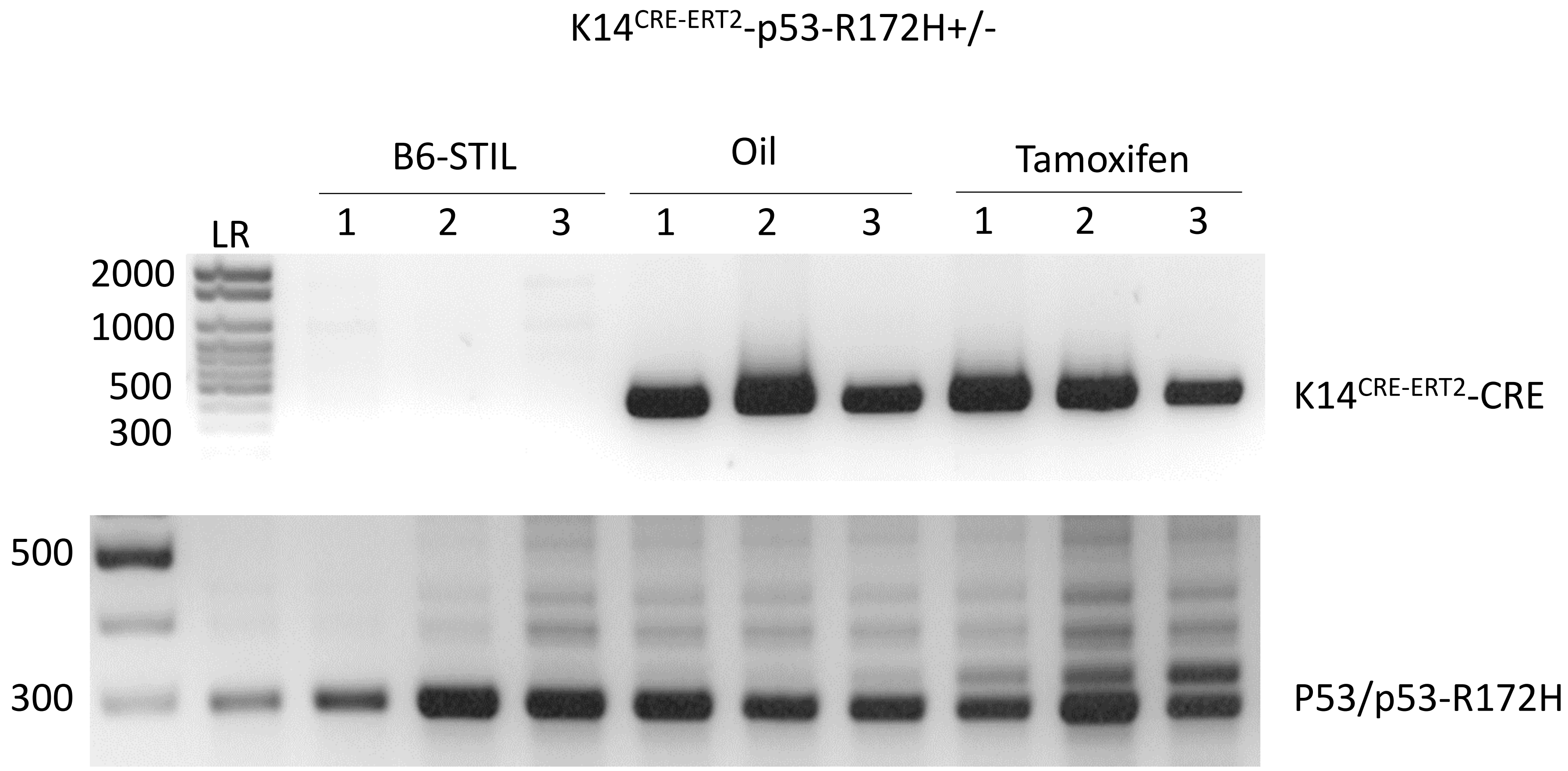

LR = LowRanger 100 bp DNA Ladder (Norgen Biotek Corp.)
