# Supplementary material for: STIL overexpression shortens lifespan and reduces tumor formation in mice
Source: PLoS Genet. 2024 Oct 28;20(10):e1011460. doi: 10.1371/journal.pgen.1011460 (PMC11542878; doi:10.1371/journal.pgen.1011460)
Supplement: Attachment — Submitted filename: 142356_1_rebuttal_3018015_sd9fdb.docx [file pgen.1011460.s015.docx]

**Manuscript number:** RC-2023-02289

**Corresponding author(s):** Alwin Krämer

## 1. General Statements [optional]

Dear Dr. Monaco,

Thank you for providing us with the opportunity to revise our article entitled “STIL over-expression shortens lifespan and reduces tumor formation in mice”. We have considered the reviewer comments carefully and revised the manuscript accordingly.

Importantly, it has recently been reported that – although in a DNA damage-driven context – PLK4-induced extra centrosomes does delay tumorigenesis independent from p53 function, similar to our findings with STIL overexpression reported in our manuscript (Braun et al.: Extra centrosomes delay DNA damage-driven tumorigenesis. Sci. Adv. 10: eadk0564, 2024).

As requested, please find a copy of the updated manuscript attached (including a separate version with the changes marked), plus a detailed list of responses to the individual comments.

Thank you once again for considering our submission.

Yours sincerely,

Alwin Krämer

(on behalf of all authors)

## 2. Point-by-point description of the revisions

We thank the reviewers for their time and effort to improve and clarify our manuscript. We now have addressed the reviewers’ suggestions in full on a point-by-point basis. Revisions in the manuscript file are highlighted in yellow.

***Reviewer #1 (Evidence, reproducibility and clarity (Required)):***

*Supernumerary centrosomes are observed in the majority of human tumors. In cells they induce abnormal mitosis leading to chromosome missegregation and aneuploidy. In animal models it is demonstrated that extra centrosomes are sufficient to drive tumor formation. Previous work studying the impact of centrosome amplification on tumor formation in vivo used Plk4 overexpression to drive the formation of supernumerary centrosomes. In this manuscript Moussa and co-workers from the Krämer group developed a mouse model in which centrosome amplification is triggered by the overexpression of the structural centrosomal protein STIL rather than the kinase Plk4 in order to a) assess the potential for centrosome amplification induced by STIL overexpression to drive tumor formation and b) to rule out any potential non-centrosomal related effects of the kinase Plk4 on tumor formation.
The authors show that STIL ovexrexpression in cells (MEFs) drives centrosome amplification and aberrant mitosis (Fig. 1), leading to chromosome missegregation and aneuploidy (Fig. 2). They also show that STIL overexpression is linked to reduced cellular proliferation and apoptosis (Fig 3). The authors then present in vivo experiments performed in mice. They observed that STIL expression causes embryonic lethality, microcephaly and a reduced lifespan (Fig 4). Despite increased STIL mRNA levels they do not detect elevated STIL protein levels in adult tissues except for the spleen. They do not detect significant increase of centrosome amplification or aneuploidy in animal tissues (Fig 4) and they conclude of a STIL translational shut down in most adult tissues. The authors then assess the impact of STIL overexpression on tumor formation. They observed a reduced spontaneous tumor formation despite elevated STIL mRNA levels in both healthy and tumor (lymphomas) tissues of mice overexpressing STIL. They don't detect increased centrosome amplification and aneuploidy in lymphomas from STIL overexpressing mice compared to lymphomas naturally occurring in control animals (Fig 5). Finally, they found that STIL overexpression suppresses chemical skin carcinogenesis using a combination of tamoxifen induction of STIL in the skin with DMBA/TPA carcinogenic treatment (Fig 7). They link this effect to an increased number of centriole and a reduction in cycling cells number in the skin of STIL overexpressing mice (Fig 6).

The manuscript is written in a clear manner. The experimental approaches are properly designed and the experimental methods are described in sufficient details. Most of the experimental data present a good number of replicates. The figures are generally well assembled despite some errors in a few panels/legends (see major and minor points). Most of the conclusions are supported by the experimental data. However, a few specific points or interpretations are not convincingly supported by the experimental data (see major points) and will need to be revised and/or reformulated.* 

***Major points:***

***1. Figures 1D and F show that MEFs hemizygous (CMV-STIL+/-) and homozygous (CMV-STIL+/+) for STIL present similar level of centrosome amplification and aberrant mitosis. Although, despite these similarities the homozygous MEFs display about two time more micronuclei and chromosomes aberrations (Fig. 2). The authors explain this discrepancy by the fact that MEFs homozygous for STIL have reduced proliferation and an increased propension to stay in interphase compared to hemizygous MEFs (Fig. 3). I don't understand why an interphase arrest would lead to a higher chromosomal instability resulting in higher micronuclei formation and abnormal karyotypes since those phenotypes are the consequences of abnormal mitosis occurring in cycling cells. I would rather argue that Homozygous MEFs are more prone to cell cycle arrest because of mitotic errors, but those mitotic errors cannot be explained by the centrosome status or the mitotic figures quantified in homozygous MEFs. Therefore, the authors explanation written as: "Graded inhibition of proliferation and accumulation of cells in interphase explains why CMV-STIL+/- and CMV-STIL+/+ MEFs contain increasing frequencies of micronuclei and aberrant karyotypes (Fig. 2) despite similar levels of supernumerary centrosomes" is not right for me. The authors should reformulate this section of the manuscript so their conclusion fit their data. The differences between hemi and homozygotes MEFs regarding chromosome stability could come from mitotic errors they did not spot using fixed immunofluorescence images of mitotic MEFs. Thus, as an optional additional experiment, analyzing live mitosis of MEFs could potentially help reconciliate results from mitotic figures and from karyotypes.***

We basically agree with the reviewer and have therefore reanalyzed our data on centriole numbers in a time-dependent manner. As already shown in Figure 3L of the initial manuscript version, the number of both CMV-STIL^+/-^ and CMV-STIL^+/+^ MEFs with supernumerary centrioles increases with passaging from passage 3 (p3) to p6. Also, in this experiment amplified centrioles were more frequent in CMV-STIL^+/+^ compared to CMV-STIL^+/-^ MEFs in both passages (p3 and p6) analyzed. We have therefore now pooled the data and substituted the former Figure panel 1D by these combined results. As the results of Figure 1F and especially those for the CMV-STIL^+/+^ MEFs had to rely on very low mitotic figure counts, because these cells only very rarely divide (as shown in Figure 3A; mitosis frequency of CMV-STIL^+/+^ MEFs 0.12%), we have now deleted Figure panel 1F from the manuscript. For the same reason - an extremely low proliferation and division rate of especially CMV-STIL^+/+^ MEFs - live cell imaging to detect different types of mitotic errors, is unfortunately not feasible.

***2. Figure 5 panel F does not support the claim of the main text and does not match the legend of the figure: In the text the authors wrote: "Ki67 immunostaining revealed that, ..., proliferation rates were elevated independent from lymphoma genotypes". If the authors claim and increased cell proliferation in lymphoma compared to lymph nodes, which is expected, they should show the data for the lymph node in the graph. In addition, in the legend the authors mentioned a "Percentage of Ki67-positive cells in healthy spleens and lymphomas from mice with the indicated genotypes." Since there are three genotypes and two tissue types but the figure presents a graph with only three bars did the Spleen and lymphoma data were combined? Or did some data were not inserted in the graph? Thus, since the data does not support the claim for an increased cell proliferation in lymphoma, the authors explanation for the increased protein level observed in these lymphomas (Fig. 5 panel E) is not supported. Therefore, the authors need to present the correct data in the figure or to change their conclusion. They will also need to correct the figure legend and to add a panel with images illustrating the Ki67 labelling in the different tissues in the figure.***

We apologize for this mistake and have corrected the legend to Figure panel 5F, which now reads: “Percentage of Ki67-positive cells in two B6-STIL, two CMV-STIL^+/-^ and one CMV-STIL^+/+^ lymphoma. For comparison, frequencies of Ki67-positive cells in healthy lymph nodes from B6-STIL mice are displayed. Data are means ± SEM from at least two independent immunostainings per lymphoma or healthy lymph node. P-values were calculated using the one-way ANOVA with post-hoc Tukey test for multiple comparison. For space reasons, only statistically significant differences are displayed”.

We agree with the reviewer that for comparison Ki67 immunostainings of healthy lymph node tissue was missing in the graph and have therefore added this information to the figure panel, which shows increased proliferation of lymphoma compared to normal lymph node cells. Also, a panel with images illustrating Ki67 labelling in healthy lymph node and lymphomas from different genotypes has been added to the figure (panel 5G).

***Minor points:***

***1. In the introduction, page 4 paragraph 3, the authors wrote: "To assess the impact of centrosome amplification on CIN, senescence, lifespan and tumor formation in vivo without interfering with extracentrosomal traits,..." they need to clarify what they meant by extracentrosomal traits.***

As requested by the reviewer we have modified the respective sentence, which now reads: “To assess the impact of centrosome amplification on CIN, senescence, lifespan and tumor formation *in vivo* with an orthologous approach without interfering with PLK4, we generated transgenic mouse models overexpressing the structural centrosome protein STIL, …”.

***2. In the 1st paragraph of the results, page 4, the authors wrote: "leads to ubiquitous transgene expression at levels similar to the CAG promoter used in most..." but there is no link to a figure presenting the mRNA levels in those mice (potentially Fig. 4F and Fig. S6). Also, in the references cited for comparison, to my knowledge, there was no measurement of Plk4 mRNA levels in tissues in the work from Marthiens and colleagues, in this work the authors assess the expression of the Plk4 transgene by investigating the presence of the protein.***

To show STIL transgene expression levels in our system, we have now linked Figure panels 1A (STIL mRNA expression in MEFs), 1B (STIL protein expression in MEFs) and Supplemental Fig. S2 (Supplemental Fig. S6 of the previous manuscript version showing STIL mRNA levels in healthy mouse tissues) to this statement as suggested. In the references now cited for comparison (Kulukian et al. 2015; Vitre et al. 2015; Sercin et al. 2016) PLK4 transgene mRNA (Kulukian et al. 2015; Sercin et al. 2016) and protein levels (Vitre et al. 2015) are shown.

***3. Page 5 second line the authors wrote: "Despite the graded increase in Plk4 expression, CMV-STIL+/- and, CMV-STIL+/+ MEFs exhibited a similar increase in supernumerary centrioles". The authors must meant increase in STIL expression or do they have data not shown about an increase of Plk4 expression? Then they explain this absence of difference in supernumerary centriole by the ability of "excess Plk4" to access the centrosome, again they probably meant STIL. Regarding this point and related to Major Point 1 it might be worth for the authors to quantify actual extra centrosomes in mitosis rather than cells with more than 4 centrioles in interphase (as in Fig. 1C, D). They might find differences in the number of centrosomes in hemizygous versus homozygous MEFs.***

We indeed meant STIL instead of PLK4 and have corrected the mistake. As described in our response to the reviewer’s major point 1 we have now reanalyzed our data on centriole numbers in a time-dependent manner. As already shown in Figure 3L of the initial manuscript version, the frequency of both CMV-STIL^+/-^ and CMV-STIL^+/+^ MEFs with supernumerary centrioles increases with passaging from passage 3 (p3) to p6. Also, in this experiment amplified centrioles were more frequent in CMV-STIL^+/+^ compared to CMV-STIL^+/-^ MEFs in both passages (p3 and p6) analyzed. We have therefore now pooled and substituted the former Figure panel 1D by these combined results.

***4. Page 5, in the first paragraph the authors mention "the rate of respective mitotic aberrations..." without defining the mitotic aberrations. For instance, in panel 1E a metaphase with 4 centrosomes is shown for CMV-STIL+/- while an anaphase with an unknown number of clustered centrosomes is presented for CMV-STIL+/+. Classifying the different types of aberrant mitotic figures (i.e: multipolar anaphases versus bipolar with clustered centrosomes) might help the authors identify differences between hemi and homozygous MEFS that may explain the differences in the proportions of chromosomes aberrations they present in Fig. 2.***

As described in our response to the reviewer’s major point 1 the number of mitotic figures that could be analyzed was extremely low, especially for CMV-STIL^+/+^ MEFs, which do only rarely divide (mitosis frequency of CMV-STIL^+/+^ MEFs 0.12%). Therefore, although certainly of value, classification of different types of mitotic aberrations is unfortunately not feasible.

***5. In Fig 4A the number of mice analyzed should be mentioned.***

After mating of B6-STIL transgenic animals with CMV-CRE mice and further breeding of successive generations, we obtained a total of 198 pups over four generations, 162 of which were born alive: 116 B6-STIL wildtype animals, 27 CMV-STIL^+/-^ and 19 CMV-STIL^-/-^ mice. We have now added these numbers to the figure legend.

***6. In Fig. 5E, the band corresponding to STIL protein is difficult to visualize in the B6-STIL control, it is therefore difficult to compare its level to the level of STIL protein in the CMV-STIL hemizygotes and homozygotes. If possible, it would improve the manuscript to present a blot with clearer results.***

We have tried to improve the quality by repeating the Western blot. Due to the small size of healthy mouse lymph nodes, resulting in low protein yields, only lysates from lymphomas were left, and these were of poor quality with a high lipid content. We therefore tried to delipidate the lymphoma lysates and hope that the result of the new blot is now somewhat clearer. Due to the low lymphoma frequency in CMV-STIL hemizygotes and homozygotes (only 2 in each case) we were unfortunately not able to prepare fresh lysates.

***7. Related to Figure 6B the authors wrote a "5 to 10 fold-increased expression..." in the text while panel 6B show a maximum of 8 fold increase.***

The respective statement has been rephrased according to the reviewer´s suggestion.

***Reviewer #1 (Significance (Required)):*** 
*Centrosome amplification is a demonstrated cause of genomic instability and tumor development as shown in multiple previous work performed in mice. In this work, Moussa and co-workers developed a mouse model that does not depends on Plk4 to trigger centrosome amplification but which depends on the overexpression of the centrosome structural protein STIL. This effort is welcome as previous works could not formally rule out potential role of Plk4, not related to its centrosome duplication function, on tumor formation. The authors show that their system is functional in MEFs where STIL overexpression drives centrosome amplification and aneuploidy. Unfortunately, in vivo, despite elevated level of STIL mRNA they do not detect centrosome amplification in tissues and consequently, they do not observe an increase rate of aneuploidy and tumor formation. This result is not surprising as previous studies using strong promoters (comparable to the one used to drive STIL expression in this study) to induce Plk4 overexpression led to similar results, i.e. an absence of centrosome amplification in adult tissues and no effects on tumor formation.
Therefore, the results and the concepts proposed in this work are not novel but they reinforce previous studies showing the deleterious effect of high level of centrosome amplification on cells. This work also confirms that strong mechanisms, here the authors propose a translational shut-down, are preventing the apparition or the persistence of high level of centrosome amplification in animal tissues. By complementing existing results with the use of an alternate experimental approach this study will be of interest for the scientific community working on the basic biological mechanisms driving aneuploidy and tumor development.*

***Reviewer #2 (Evidence, reproducibility and clarity (Required)):***
*In this manuscript, Moussa et al. describe the effects of over-expressing the centriole duplication factor STIL in whole mice and with expression restricted to the skin. They find that over expression of STIL, similar to that of PLK4, induces centriole overduplication, abnormal mitoses, and genetic instability leading to cell arrest. Additionally, over-expressing STIL results in microcephaly, perinatal lethality and a shortened lifespan. In addition, they do not find that expression of the p53 R127H mutant alleviates the cell growth defect. Moreover, overexpression of STIL does not lead to increased general tumour formation and suppresses tumour formation in an induced skin tumour model.

Although this is an interesting manuscript, the authors need address a number of issues before this manuscript can be recommend the manuscript for publication. Importantly, the manuscript lacks statistical analyses to support some of their conclusions, some figures should be quantified, and controls are missing in some cases.* 

***Major Issues***

***1. Many of the figure panels lack appropriate statistical analyses to support the conclusions (see details below). This needs to be rectified.***

In view of the limited number of mice (due to an increased frequency of pups that died around birth) and the resulting impossibility of performing several (>3) independent experiments in many cases, we have decided to limit the statistics in the main text to a descriptive analysis without mentioning inferences (p-values). Nevertheless, we have now included the missing statistical analyses in the figure panels and/or legends. However, the reported p-values (*p≤0.05, **p≤0.01, ***p≤0.001; ns, not significant) should be interpreted as descriptive rather than confirmatory values.

***2. The authors suggest that the interpretation of PLK4 over-expression studies are hampered by the possibility of centriole/centrosome independent PLK4 roles and that STIL overexpression circumvents some of these issues. Although orthologous approaches to problems are always desired, STIL itself has also been implicated in other cellular processes, such as the Sonic hedgehog pathway (Carr AL, 2014) and in cell motility (Liu Y, 2020). In addition, the data presented in the manuscript are suggestive of a STIL function in the mouse that is independent of centriole number. The authors demonstrate that the amount of centriole over-duplication in MEFs containing a single copy of the STIL over-expression locus is equivalent to that of MEFs carrying two copies. However, in most other assays, the homozygous lines display more severe phenotypes, suggesting that STIL might have a function outside centriole duplication. The authors need to discuss this further in a revised manuscript.***

As described in our response to major point 1 and minor point 3 of reviewer 1 we have now reanalyzed our data on centriole numbers in a time-dependent manner. As already shown in Figure 3L of the initial manuscript version, the number of both CMV-STIL^+/-^ and CMV-STIL^+/+^ MEFs with supernumerary centrioles increases with passaging from passage 3 (p3) to p6. Also, in this experiment amplified centrioles were more frequent in CMV-STIL^+/+^ compared to CMV-STIL^+/-^ MEFs in both passages (p3 and p6) analyzed. We have therefore now pooled the data and substituted the former Figure panel 1D by these combined results, which show that, similar to other models, also regarding STIL overexpression the homozygous line displays a more severe phenotype, which does therefore per se not argue for a STIL function outside the centrosome. However, as a few recent studies indeed suggest additional roles of STIL, we have amended the respective passages in the revised version of the manuscript accordingly.

***3. Why did the authors use the p53 R127H mutant instead of a p53 knockout or null allele system? The R127H mutant has a gain-of-function phenotype and cells expressing this mutant display different phenotypes than a p53 null. The primary conclusion in one of the references cited by the authors (Caulin C, 2007) is that p53R127H is a gain-of-function mutant and behaves distinct from loss-of-function p53 mutations, such as deletions using floxed alleles. Throughout the manuscript, the authors use terms that suggest the R127H allele is equivalent to a loss of function mutant. Given that supernumerary centriole growth arrest is universally suppressed by inactivation of p53 it is somewhat surprising that this pathway is not active in response to STIL over-expression. The authors should confirm this key conclusion by depleting p53 in MEFs using RNAi, or by using mice where complete inactivation of p53 can be achieved.***

We agree with the reviewer that the p53-R172H mutant version of p53 is not equivalent to a p53 knockout. We have therefore and as suggested by reviewer 3 as well (see also our response to point 3 of reviewer 3) corrected the wording and have substituted “absence of p53” by “interference with p53 function” where appropriate. In addition, we now have added data to the manuscript, which show that neither p53 expression nor p53-S18 phosphorylation becomes induced during prolonged cultivation and passaging of CMV-STIL transgenic MEFs (see Figure 3B of the revised manuscript). Importantly, this finding is in line with a recent report showing that PLK4-induced extra centrosomes may not rely on p53 for tumor suppression and cell death induction (Braun et al.: Extra centrosomes delay DNA damage-driven tumorigenesis. Sci. Adv. 10: eadk0564, 2024). Similarly, it has been recently shown that centrosome amplification increases apoptosis independently of p53 in PLK4-overexpressing cells treated with DNA-damaging agents (Edwards et al.: Centrosome amplification primes for apoptosis and favors the response to chemotherapy in ovarian cancer beyond multipolar divisions. *bioRxiv* 2023.07.28.550973, 2023). Therefore, these findings and references have now been added to results and discussion sections of the revised manuscript.

A plethora of p53-related findings in mouse models, including the majority of results on PLK4-induced tumor formation in mice, is based on p53 knockouts, a situation that is only rarely found in human cancers. In contrast, the p53-R172H missense mutation in mice corresponds to the p53-R175H mutation in human tumors, which has the highest occurrence in diverse human cancer types among all p53 hotspot mutations, and results in a transcriptionally inactive protein that accumulates in cells, similar to the majority of naturally occurring versions of mutant p53 (Yao et al.: Protein-level mutant p53 reporters identify druggable rare precancerous clones in noncancerous tissues. Nat Cancer 4: 1176-1192, 2023; Chiang et al.: The function of mutant p53-R175H in cancer. Cancers 13: 4088, 2021). We therefore believe that it more faithfully recapitulates the situation in p53-mutant tumors than a p53 knockout.

Although basically an important and valid experiment, depleting p53 in STIL-transgenic MEFs using RNAi is not easily done as (i) transfection of MEFs per se is difficult and (ii) STIL-overexpressing MEFs do only slowly proliferate and are prone to senescence and apoptosis (see Figure 3), all phenotypes which are even further exacerbated after transfection. Generation of STIL-transgenic mice with complete inactivation of p53 on the other hand is an extremely time-consuming endeavor that would lead to a significant delay of publication of our results. Given that currently similar data are published by other groups (Braun et al.: Extra centrosomes delay DNA damage-driven tumorigenesis. Sci. Adv. 10: eadk0564, 2024; Edwards et al.: Centrosome amplification primes for apoptosis and favors the response to chemotherapy in ovarian cancer beyond multipolar divisions. *bioRxiv* 2023.07.28.550973, 2023), we do not think that this would be appropriate.

***Minor Issues and details***

***Figure 1
1. Panel E. It is unclear what the authors are calling an 'aberrant mitosis'. Typically an aberrant mitosis refers to chromosomal abnormalities such as multipolar spindles, anaphase bridges or micronuclei (which they quantify in Figure 2).***
The aberrant mitotic figures presented in Figure 1E show a clustered metaphase with 4 centrosomes (2 per pole; 2 centrioles per centrosome) for CMV-STIL^+/-^ MEFs and a clustered telophase with 2 centrosomes (1 per pole; 5 centrioles per centrosome) for CMV-STIL^+/+^ MEFs. This is now specified in detail in the legend to Figure 1E.

***2. Panel E. Please include images representing a normal mitosis from control cells derived from B6-STIL mice.***

As suggested, we have now included a representative image of a normal mitosis from B6-STIL control mice.

***Figure 2
1. Panels B, E and F. Statistical significance is not indicated between B6-STIL and CMV-STIL+/- or CMV-STIL+/- and CMV-STIL+/+. The authors indicated a 'graded' phenotype which is qualitatively apparent, but should be backed by statistical analysis.***

We have now included a statistical analysis. However, and as already described in our answer to major issue 1 of this reviewer, the reported p-values should be interpreted as descriptive rather than confirmatory values due to the limited number of independent experiments.

***2. Can the authors indicate how they scored a tetraploid cell? Some of the cells are 100% tetraploid while others contain other aberrations.***

According to the International System for Human Cytogenomic Nomenclature (ISCN) version from 2020, polyploidy is defined by the modal numbers of chromosomes in the karyotype. A number of 81-103 chromosomes is called near-tetraploid, at which a hypotetraploidy (81-91 chromosomes) is distinguished from a hypertetraploidy (93-103 chromosomes) (An International System for Human Cytogenomic Nomenclature, Karger (2020), Eds.: McGowan-Jordan, Hastings, Moore). For mouse karyotypes respective numbers were recalculated on the basis of a diploid chromosome content of 40 instead of 46 chromosomes. To be strictly in accordance with this nomenclature, we have exchanged the term "tetraploid" by "near-tetraploid".

***3. Is the height of the rows in Panel D significant? What are the solid black rows?***
We thank the reviewer for this comment/observation. We have now increased the resolution of this part of the figure. Unfortunately, the resolution had deteriorated so much when the pdf file was created that individual lines were no longer recognizable. The height of the lines should be identical, as single lines correspond to the karyotypes of each metaphase cell analyzed, while chromosomes are plotted as columns. The solid black lines separate independently established MEF lines with the indicated STIL genotypes from each other. At least 20 metaphase cells per MEF line were analyzed. We have now explained these points in the figure legend.

***Figure 3
1. Panels C, F, G, and K require statistical analyses.***

We have now included the appropriate statistical analyses in the figure panels and/or legends. However, the reported p-values should be interpreted as descriptive rather than confirmatory values due to the limited number of independent experiments.

***2. Panel D should be quantified.***

We have now included a quantification of the protein bands in panels B, E (former panel D), and K of the revised manuscript and explained the quantification procedure in detail in the methods section.

***3. Panel E. mRNA expression is quantified in RPKM here, while GeTMM is used in Figures 3I and Supplementary Figures S2 and S6. Is there a reason this panel uses a different method? RPKM can be used for intra-sample comparisons, but is not ideal for comparison among different samples.***

We now uniformly quantify mRNA expression in GeTMM in all figures of the revised manuscript version as requested.

***4. Panel G. Can the authors show the original FACS profiles in Supplementary material?***

As requested, we have now included representative examples of original FACS profiles from the cell cycle analyses into Supplemental Figure S5.

***5. Panel H. Requires molecular weight markers***

Molecular weight markers for the DNA ladder (L) with the corresponding bp size have now been included into the Figure panel (formerly 3H, 3I in the revised version of the manuscript).

***6. Panel J. Missing B6-STIL control. Quantify Western blots.***
We have now included an immunoblot showing STIL protein expression levels in passage p1-p5 of B6-STIL control MEFs as well as a quantification of the protein bands into the Figure panel (formerly 3J, 3K in the revised version of the manuscript). The quantification procedure has been explained in detail in the methods section of the revised manuscript version.

***Figure 4
1. The authors mention 'Simultaneously, we found an increased frequency of pups that died around birth.' Can the data for this be included?***

After mating B6-STIL transgenic animals with CMV-CRE mice and further breeding of successive generations, we obtained a total of 198 pups over four generations, of which 162 were born alive: 116 B6-STIL wildtype animals, 27 CMV-STIL^+/-^ and 19 CMV-STIL^+/+^ mice. We have now added these numbers to the figure legend. Stillbirths increased over the generations: while in the first generation after mating B6-STIL animals with CMV-CRE mice all pups (B6-STIL wildtype animals and STIL heterozygotes) were born alive, in the fourth generation (from mating CMV-STIL transgenic mice with each other) 54% of the pups were stillborn. We have now included this observation into the main text to further emphasize the impact of STIL overexpression on perinatal lethality.

***2. Panels B and D. Please include the data for CMV-STIL+/-.***

We now have included a representative H&E-stained histological section of a CMV-STIL^+/-^ mouse brain into Figure panel 4D as suggested by the reviewer. For space reasons we have not added an extra image of a CMV-STIL^+/-^ total brain into Figure panel 4B, as this does not add novel information.

***3. Panels C, F and K require statistics.***

As requested, we have now included the appropriate statistical analysis in the figure panels and/or legends. However, the reported p-values should be interpreted as descriptive rather than confirmatory values due to the limited number of independent experiments.

***4. Panel F. Include statistical analysis.***

We have now included the appropriate statistical analysis in the figure panels and/or legends. However, the reported p-values should be interpreted as descriptive rather than confirmatory values due to the limited number of independent experiments.

***5. Panel G/H. The levels of STIL in the CMV-STIL+/+ spleen are higher than the other samples, yet there is no concomitant increase in centriole overduplication. Can the authors comment on this?***

Interestingly, we indeed found a higher STIL protein expression level in spleen tissue from CMV-STIL^+/+^ as compared to B6-STIL control and CMV-STIL^+/-^ mice. Nevertheless, the amount of splenocytes with supernumerary centrioles was only marginally increased in these animals. A similar finding has recently been described for B lymphocytes with upregulated PLK4 expression after PLK4 transgene induction by exposure to doxycycline in vivo (Braun et al.: Extra centrosomes delay DNA damage-driven tumorigenesis. Sci. Adv. 10: eadk0564, 2024). Here, the lack of B cells with supernumerary centrioles despite increased PLK4 levels was explained by increased apoptosis and thereby selection against and rapid loss of PLK4-overexpressing cells. In line, we show that CMV-STIL^+/+^ MEFs have increased rates of senescence and apoptosis (Fig. 4).

***6. Panel J. The font within the plots is difficult to read.*** 
We thank the reviewer for this comment/observation. We have now increased the resolution of this figure panel, and the font is now outside of the plots.

***Figure 5
1. Panels B, D and G require statistics.***

We have now included the appropriate statistical analysis in panel B and G. However, the reported p-values should be interpreted as descriptive rather than confirmatory values due to the limited number of independent experiments. No further statistical analysis can be done for panel D as in some cases (lymph node from B6-STIL mouse, lymphoma from CMV-STIL^+/+^ mouse) only one measurement exists.

***2. Panel F. The legend indicates that these data are from spleens and lymphomas. Is this correct? Would the results from non-lymphoma cells in the spleen mask the results from lymphoma cells?***

We apologize for this mistake and have corrected the legend to Figure panel 5F, which now reads: “Percentage of Ki67-positive cells in two B6-STIL, two CMV-STIL^+/-^ and one CMV-STIL^+/+^ lymphoma. For comparison, frequencies of Ki67-positive cells in healthy lymph nodes from B6-STIL mice are displayed. Data are means ± SEM from at least two independent immunostainings per lymphoma or healthy lymph node. P-values were calculated using the one-way ANOVA with post-hoc Tukey test for multiple comparison. For space reasons, only statistically significant differences are displayed”.

***3. Panel F. The authors indicate that 'In line, assessment of lymphomas from B6-STIL control, CMV-STIL+/- and CMV-STIL+/+ mice by Ki67 immunostaining revealed that, corresponding to STIL protein levels, proliferation rates were elevated independent from lymphoma genotypes'. However, Ki67 levels, the marker for proliferation actually decreased in these samples indicating less proliferative cells. This needs to be clarified since the data shown appears to show the opposite of what is stated in the mansucript....***

As noticed by the reviewer further below, differences in the percentages of Ki67-positive, proliferating cells between lymphomas from B6-STIL, CMV-STIL^+/-^ and CMV-STIL^+/+^ mice were statistically not significant. However, we have now for comparison added the results of Ki67 immunostaining of healthy lymph node tissue to Figure panel 5F, which show increased proliferation of lymphoma compared to normal lymph node cells. Also, a panel with images illustrating Ki67 labelling in healthy lymph node and lymphomas from different genotypes has been added to the figure (panel 5G). These data reveal that, independent from the genotype, proliferation rates of lymphoma cells are increased as compared to healthy lymph nodes, thereby further corroborating our assumption that STIL protein levels in lymphomas are increased as a consequence of their increased proliferation and independent from STIL transgene expression.

***4. Corresponding to point 3 above, the authors suggest that 'STIL protein expression is a consequence of increased lymphoma cell proliferation.' This hypothesis cannot explain STIL protein levels if proliferation has actually decreased.***

Please see our response to point 3 above.

***5. Corresponding to point 3 and 4 above, the actual data is marked as non-significant indicating there is actually no proliferative difference among the samples.***

This is correct. See also our comments to point 3 and 4 above.

***6. Panel 5I. The authors state that 'On the other hand, overall levels of chromosomal copy number aberrations were higher in lymphomas (mean gains + losses: 225.2 Å} 173.7 Mb) as compared to healthy tissues (mean gains + losses: 87.3 Å} 127.5 Mb; p=0.06), irrespective of their STIL transgene status (Fig. 4J; Fig. 5I), although the difference did not quite reach statistical significance.' The authors need to soften this statement since statistically, the samples are not different. For example, 'On the other hand, overall levels of chromosomal copy number aberrations appeared to trend higher in lymphomas as compared to healthy tissues irrespective of their STIL transgene status, although the difference did not quite reach statistical significance.'***
The statement was rephrased according to the reviewer´s suggestion.

***Figure 6
1. Panels A, B, and C require statistical analysis.***

We have now included the appropriate statistical analyses into panels A, B, and C in the figure panels and/or legends. However, the reported p-values should be interpreted as descriptive rather than confirmatory values due to the limited number of independent experiments.

***2. The figure legend references to panels C and D appear to be swapped.***

We thank the reviewer for this comment/observation. We have corrected this mistake.

***3. Panel F. Indicate that the samples are not significantly different.***

We have now included the appropriate statistical analysis including the indication that the samples are not statistically significantly different.

***4. Corresponding to point 3, the authors indicate that 'the proportion of Ki67-positive cycling cells was lower in tamoxifen-treated... ... although the difference did not quite reach statistical significance.' The authors need to soften this statement to reflect that the samples are not statistically different (i.e. 'appeared lower' or similar).***
The statement was rephrased according to the reviewer´s suggestion.

***Figure 6 and 7 
Do you have data for B6-STIL animals treated with and without tamoxifen? The experiments as shown demonstrate the differences between control and tamoxifen-treated animals of the same genotype, but it is unclear if any of these effects are due to the underlying genotypes or from tamoxifen itself.*** 
The experiments presented in Figures 6 and 7 have not been performed in B6-STIL control mice with and without tamoxifen treatment.

***Supplemental Figure 1
1. Please include molecular weight marker for this and all panels showing PCR products.***

Molecular weight markers for the DNA ladder (L) with the corresponding bp size have now been included into all Figure panels showing PCR products as requested.

***2. The B6-STIL and CMV-STIL+/- lines should contain a larger MW band corresponding to the STIL-F and STIL-R PCR product. Please show if possible.***

We thank the reviewer for the important remark. We agree that there should be a large PCR product band at around 3000 bp containing the bacterial neomycin phosphotransferase gene (TK-neo-pA) and the STOP cassette in the B6-STIL control mice/MEFs, and two PCR product bands (large: 3000 bp, small: 410 bp) in the heterozygous CMV-STIL^+/-^ mice/MEFs. When we began with genotyping, we did indeed observe both bands depending on the STIL background (see figure below). However, the band intensity of the larger PCR product was relatively weak (arrowheads) compared to the smaller PCR product, and its visibility was dependent on genomic DNA input and PCR efficiency. During the PCR optimization process, the PCR conditions were changed in such a way that the yield of the small band were increased despite small input amounts of genomic DNA, but at the expense of the large PCR product band (arrows). At the end of the optimization process the larger PCR product had almost disappeared, making the discrimination between heterozygous CMV-STIL^+/-^ and homozygous CMV-STIL^-/-^ DNA difficult. Therefore, we decided to additionally check for STOP cassette excision in a second PCR approach in parallel. In the genotyping results shown in Supplemental Figure S1B, which have been produced after PCR optimization, no larger STIL PCR product band was visible anymore.

***Supplemental Figure 6 
1. The 'Spleen' sample is missing the B6-STIL control data. 'Liver' is missing CMV-STIL+/+. Please include or indicate why they are missing. The plot order of the samples differs for 'Liver' (red, black) compared to the others (black, red, blue). Indicate statistical significances.*** 
We apologize for this mistake, have corrected the Figure (formerly Supplemental Figure S6, S2 in the revised version of the manuscript), and have included the missing spleen and liver samples.

***General issues 
1. The materials and methods indicate that HPRT and PIPB were used as reference genes, but only HPRT is referred to in the qPCR figure legend.***

We thank the reviewer for this comment/observation. As generally recommended (Vandesomele et al., Genome Biol 3(7): research0034.1-research0034.11, 2002; Kozer and Rapacz, J Appl Genet 54(4): 391-406, 2013) we used both reference genes for accurate normalization of qPCR in all experiments. We have now corrected this mistake in the figure legend.

***2. Figure panels 1F and 3C display 95% confidence intervals while others use SEM. Is there a reason for this?***

In the two referenced figures (former Figure 1F has been deleted from the manuscript, see also our comment to point 1 of reviewer #1 for reasons; Figure 3C of the former manuscript is now Figure 3D in the revised manuscript version) the endpoint variable was defined by whether individual cells in a single experiment showed a certain property or not (binary variables). By definition, these kinds of variables show a nonsymmetric error structure, which cannot be expressed properly by a single value such as the standard error (SEM), but can be covered correctly by a confidence interval. For the same reason, Fisher’s exact tests were employed to obtain p-values in these situations. In the other figures, the relevant endpoint variables were roughly normally distributed, either directly, or due to them being an average of many values. In this case, a symmetric SEM was thus considered sufficient, and t-tests were used for p-values. To make this clear in the figures, we used different display options to distinguish between error bars showing SEM or 95% CI.

***Reviewer #2 (Significance (Required)):*** 
*In this manuscript, Moussa et al. describe the effects of over-expressing the centriole duplication factor STIL in whole mice and with expression restricted to the skin. They find that over expression of STIL, similar to that of PLK4, induces centriole overduplication, abnormal mitoses, and genetic instability leading to cell arrest. Additionally, over-expressing STIL results in microcephaly, perinatal lethality and a shortened lifespan. In addition, they do not find that expression of the p53 R127H mutant alleviates the cell growth defect. Moreover, overexpression of STIL does not lead to increased general tumour formation and suppresses tumour formation in an induced skin tumour model. Although this is an interesting manuscript, the authors need address a number of issues before this manuscript can be recommend the manuscript for publication. Importantly, the manuscript lacks statistical analyses to support some of their conclusions, some figures should be quantified, and controls are missing in some cases.* 

***Reviewer #3 (Evidence, reproducibility and clarity (Required)):*** 
*Previously it has been proposed that supernumerary centrioles play important deleterious effects in vivo including increased tumorigenesis. However, the work was inconclusive because the way of inducing centriole amplification via the PLK4 kinase could have induced other effects besides supernumerary centrioles. To resolve this question, the authors generated a mouse model of centrosome amplification, in which the structural centriole protein STIL is overexpressed. Using this mouse model in vivo along with mutant mouse embryonic feeder (MEF) lines in vivo, the authors test out the role of centrosome amplification in vivo in animal development, lifespan, and tumorigenesis. They report both embryonic lethality, defects in brain development, and shortened life span in these mice. They also find that skin tumorigenesis is reduced in the mutant mice, and demonstrates that the STIL overexpression effects are not perturbed in a dominant negative p53 model. The authors demonstrate that STIL overexpression causes centrosome amplification accompanied by aneuploidy, which however is highly deleterious for cell fitness even in the absence of p53. Clearly, tissue corrective mechanisms lead to the elimination of cells with extra centrosomes and/or aneuploidy by impaired proliferation, senescence, and apoptosis. This finding is interesting and significant and seems worthy of dissemination to the broader readership.*

*This study is thorough and well executed and there is a significant body of work that leads to solid conclusions. The data is convincing, and the figure are well presented. It was refreshing to read this paper, as it was not so cluttered with data that the message gets murky, yet the data was clearly very substantial. The text is clear and easy to follow.*

***• There really are only minor aspects of this paper that need correction, in my opinion. The text should be thoroughly checked for typos, few extra redundant words here and there, and a couple of confusing sentences.***
As suggested by the reviewer we have rechecked the manuscript for typos, redundancies, and confusing sentences and corrected where necessary and appropriate.

***• For example, the last sentence in abstract is confusing 'These results suggest that supernumerary centrosomes... [result in]... tumor formation' because it should read 'reduced tumor formation' or 'impairs tumorigenesis' or otherwise be written more clearly because it seems to convey the opposite message the way it is right now.*** 
We thank the reviewer for this comment and have corrected the sentence, which now reads: “These results suggest that supernumerary centrosomes impair proliferation in vitro as well as in vivo, resulting in reduced lifespan and delayed spontaneous as well as carcinogen-induced tumor formation”.

***• The p53 dominant negative mutant is not exactly a KO so it is not fair to say "in the absence of p53"; the verbiage should be corrected and checked throughout the paper - perhaps 'interfering with p53 normal function' is more appropriate.***
As suggested by the reviewer we have corrected the wording and have substituted “absence of p53” by “interference with p53 function” where appropriate.

***• The sentence "Senescence- and apoptosis-driven depletion of the stem cell pool may explain reduced life span and tumor formation in STIL transgenic mice." from discussion is highly speculative and should be edited to clearly convey its speculative nature or removed entirely.*** 
We agree with the reviewer and have deleted the sentence from the discussion section of the manuscript.

***Reviewer #3 (Significance (Required)):*** 
*Clearly, tissue corrective mechanisms lead to the elimination of cells with extra centrosomes and/or aneuploidy by impaired proliferation, senescence, and apoptosis. This finding is interesting and significant and seems worthy of dissemination to the scientific community. It adds to previous work on another centriole related protein PLK4 kinase that led to very different conclusions.*
